# The Association Between Patient-Centered Communication and Primary Care Quality in Urban China: Evidence From a Standardized Patient Study

**DOI:** 10.3389/fpubh.2021.779293

**Published:** 2022-02-04

**Authors:** Min Su, Zhongliang Zhou, Yafei Si, Xiaojing Fan

**Affiliations:** ^1^School of Public Administration, Inner Mongolia University, Hohhot, China; ^2^School of Public Policy and Administration, Xi'an Jiaotong University, Xi'an, China; ^3^School of Risk & Actuarial Studies and CEPAR, University of New South Wales, Sydney, NSW, Australia

**Keywords:** patient-centered communication, primary care, quality, standardized patient, China

## Abstract

**Background:**

Effective patient-physician communication has been considered a central clinical function and core value of health system. Currently, there are no studies directly evaluating the association between patient-centered communication (PCC) and primary care quality in urban China. This study aims to investigate the association between PCC and primary care quality.

**Methods:**

The standardized patients were used to measure PCC and the quality of health care. We recruited 12 standardized patients from local communities presenting fixed cases (unstable angina and asthma), including 492 interactions between physicians and standardized patients across 63 CHCs in Xi'an, China. PCC was scored on three dismissions: (1) exploring disease and illness experience, (2) understanding the whole person, and (3) finding common ground. We measured the quality of the primary care by (1) accuracy of diagnosis, (2) consultation time, (3) appropriateness of treatment, (4) unnecessary exams; (5) unnecessary drugs, and (6) medical expenditure. Ordinary least-squares regression models with fixed effects were used for the continuous variables and logistic regression models with fixed effects were used for the categorical variables.

**Results:**

The average score of PCC1, PCC2, and PCC3 was 12.24 ± 4.04 (out of 64), 0.79 ± 0.64 (out of 3), and 10.19 ± 3.60 (out of 17), respectively. The total score of PCC was 23.22 ± 6.24 (out of 84). We found 44.11% of the visits having a correct diagnosis, and 24.19% of the visits having correct treatment. The average number of unnecessary exams and drugs was 0.91 ± 1.05, and 0.45 ± 0.82, respectively. The average total cost was 35.00 ± 41.26 CNY. After controlling for the potential confounding factors and fixed effects, the PCC increased the correct diagnosis by 10 percentage points (*P* < 0.01), the correct treatment by 7 percentage points (*P* < 0.01), the consultation time by 0.17 min (*P* < 0.01), the number of unnecessary drugs by 0.03 items (*P* < 0.01), and the medical expenditure by 1.46 CNY (*P* < 0.01).

**Conclusions:**

This study revealed pretty poor communication between primary care providers and patients. The PCC model has not been achieved, which could be one source of the intensified physician-patient relationship. Our findings showed the PCC model in the primary care settings has positive associations with the quality of the primary care. Interactions with a higher score of PCC were more likely to have a correct diagnosis and correct treatment, more consultation time, more unnecessary drugs, and higher medical expenditure. To improve PCC, the clinical capacity and communication skills of primary care providers need to be strengthened. Also, strategies on reforming the pay structure to better reflect the value of physicians and providing a stronger motivation for performance improvement are urgently needed.

## Introduction

Violence against physicians has been a serious occupational hazard and public health issue globally, as well as in China (1). The World Health Organization (WHO) reported that 8–38% of health workers suffer physical violence in their career (2). In China, the prevalence of violence against health professionals ranges from 50 to 83.3% (3, 4), which has received considerable attention (5, 6). Chinese Medical Doctor Association reported that ~60% of medical staff had experienced verbal abuse, and almost one in seven had been physically assaulted in 2015 in China (7). Such widespread violence impacts heavily on the delivery of health care services, which could include a deterioration in the health care quality, and the intention to leave the profession (8). Evidence from a study of 933 physicians in 29 public hospitals in Shandong province, 49% had reported the intention to leave the profession (9). This in turn could result in a reduction in health services available to the general population, and an increase in health costs (2). It has been proven that violence together with stress possibly accounts for about 30% of the overall health costs of illness and accidents (10). The evidence clearly shows the physician-patient relationship is far too poor in China (2, 11). Previous studies have widely documented that PCC may be helpful to relieve the patient violence against physicians (11). PCC can lead to multiple positive outcomes, such as strengthening mutual understanding, mitigating patients' uncertainty on their illness and disease experience, and empowering patients to making decisions during the health interactions between physicians and patients. That is important to facilitate harmonious physician-patient relationship, and further ensure patient trust, and reduce physician-patient violence (11–13).

Patient-centered communication (PCC) has been one of the most frequently discussed issues in health care over the past few decades (14, 15). The Institute of Medicine (IOM) defined patient-centered communication as a model that not only aims to obtain necessary information on diagnosis and treatment, but also to acquire patient's experience of presenting themselves, their problems, needs, expectation, and feelings, and to achieve better understanding and agreement between physician and patient on disease management (16–18). Effective PCC has been considered a central clinical function and core value of health systems (19). PCC is proven to achieve a variety of worthy outcomes, including patient recovery, emotional health, physical function, physiologic outcomes, better patient satisfaction, patient adherence, fewer malpractice complaints, the efficiency of healthcare, lower cost of health care, and time (16–18). For example, previous studies have showed that PCC is a strong driving force of patient trust (or rather patient trust is a mediator). When physicians communicate rapport, respect, care, and understating of patients, patient trust is achieved. When patients trust their physicians, they would be having better adherence to treatment, follow physicians' recommended behaviors, thus resulting in better health outcomes (11, 20). Evidence from U.S. Department of Health and Human Services (HHS) has indicated that PCC may contribute directly or indirectly to multiple outcomes in cancer care (e.g., adherence, prevention and early detection of cancer, accurate diagnosis, and completion of evidence-based treatment) (16). In addition, PCC is significantly important especially in primary care settings (21). Primary care is considered the first contact and gatekeeper for patients to health service and physicians should be more familiar with how diseases are prevented at early stages. Physicians who work in primary care settings are heavily influenced by patients' social context, therefore, how patients' problems being discussed, and how their needs, expectations, and feelings being considered is very important (22).

Although several studies have investigated PCC in developed countries, evidence from low and middle-income countries is scarce. Only a few studies were from China. Evidence from a study of 483 Chinese patients has documented that PCC directly improved emotional health, and patient trust positively moderated the effects of PCC and emotional health (23). Another study conducted in public teaching hospitals in Guiyang, Guizhou, China showed that patients expressed strong preferences concerning physician respect for patient perspective (24). However, there exist several research gaps. First, there was scare empirical evidence about how PCC improved the quality of diagnoses and treatments. Second, previous studies often used patient perception and recall-based surveys. Since PCC is a multifaceted concept, therefore, how to obtain objective information about communication behaviors between physician and patient is a significant challenge (18). Four main methods were commonly used to gather communication information between physician and patient: chart abstraction, clinical vignettes, self-reported survey, and SP approach (25–27). SP approach has obvious advantages over other methods: (1) SPs are unannounced to seek healthcare in a practical setting with concealed recording equipment, which could have detailed information regarding medical visit in the real-world setting; (2) SP approach is free from observation and recall bias by recording the interactions between patients and physicians; and (3) it enables comparisons across different providers. Due to these advantages, the SP approach has been regarded as the gold standard for evaluating the quality of healthcare in developed countries (28). SP approach, which focuses on direct communications between physicians and patients and measures the quality according to the standardized clinical checklist, minimizes recall bias and subjective bias (29–32).

Currently, there are no studies directly evaluating PCC and its association with the quality of primary care in China. This study aims to measure PCC in the primary care settings in urban China using the SP method and to investigate the association between PCC and the quality of primary care. We hypothesize that higher scores of the PCC are associated with significant improvements in the quality of primary care.

## Methods

### Theoretical Framework of PCC

**Figure 1:**
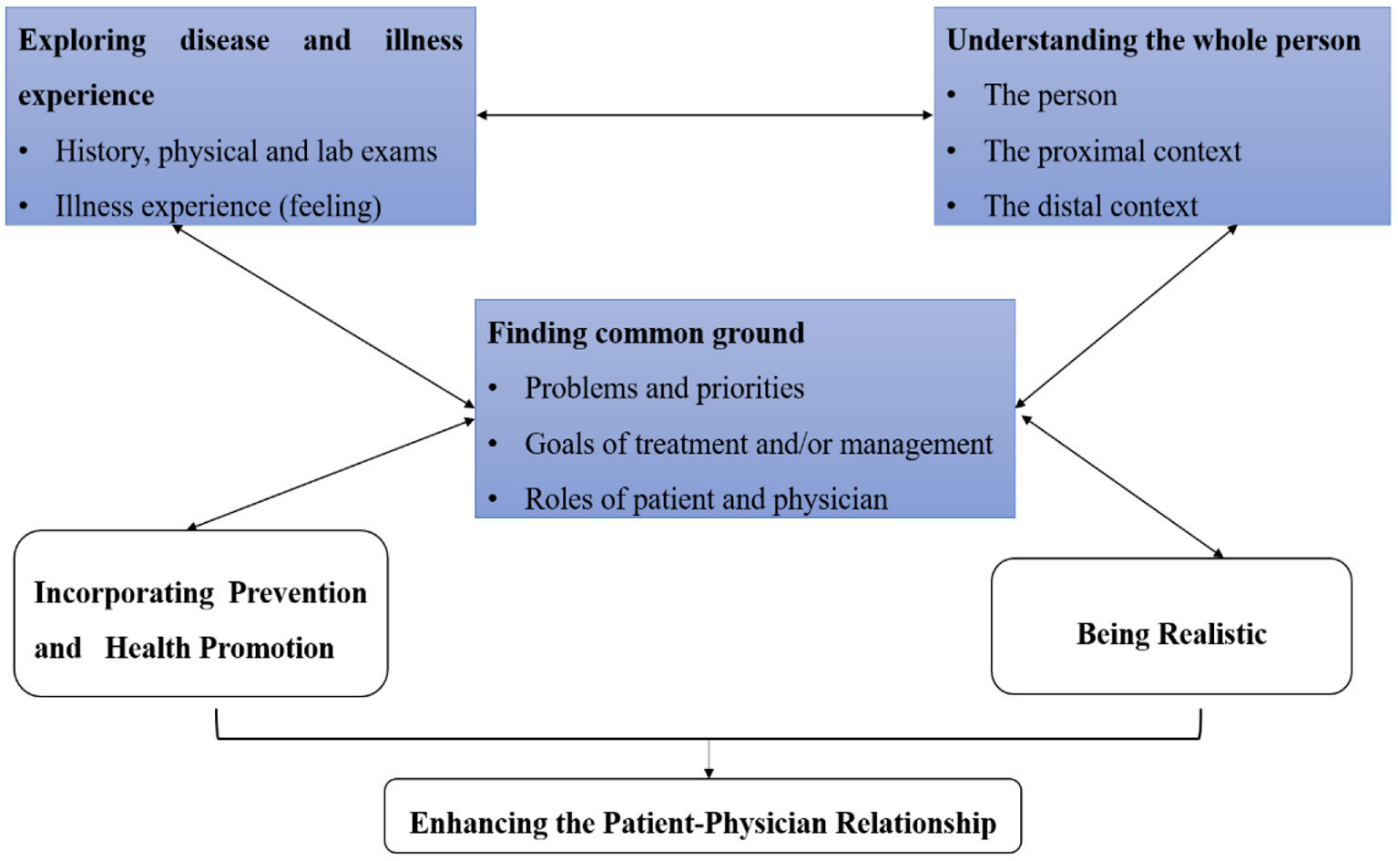
Theoretical framework of patient-centered communication (PCC). We used three dimensions to measure the process of the PCC in urban China: exploring disease and illness experience, understanding the whole person, and finding common ground. Source: Adopted from (33).

Modern medicine is a comprehensive model to describe and explore how illness is the result of the interplay of biological, psychological, and social determinants plus individual behaviors (17). The goal of this model is to develop a patient-centered care model to achieve the best health outcomes. Hurwitz et al. (29) first introduced the term “patient-centered medicine” and contrasted it with “disease-centered medicine”. Stewart (30) first developed the patient-centered clinical method (developed by the Patient-Doctor Communication Group at the University of Western Ontario). They proposed 6 dimensions to define PCC: exploring disease and illness experience, understanding the whole person, finding common ground, incorporating prevention and health promotion, improving the patient-doctor relationship, and being realistic. We used the first three dimensions to measure the process of PCC: exploring disease and illness experience, understanding the whole person, and finding common ground (Figure 1) (30). In this model, both disease and illness experience should be explored. Disease experience means physical or mental disorders, which should be explored by history taking, physical, and laboratory examinations. Illness experience refers to a patient's expectations and feelings. Understanding the whole person means physicians should understand a patient's disease and illness in the context of their life settings, including family, job, and social networks. Finding common ground refers to an agreement between physician and patient on three areas: the nature of the problem, the goals of treatment and disease management, and the roles of patient and physician.

### Study Design and Setting

A standardized patient (also known as a fake patient, or simulated patient) is a healthy individual recruited from the local communities and trained to portray an actual patient's historical, physical and emotional features in a standardized way to collect the communication information between physician and patient (31). SPs are coached to present their initial symptoms and answer any questions that the physician may ask as part of history taking, in a manner consistent with the underlying condition. We conducted the SP study in community health centers (CHCs). Two common outpatient diseases (unstable angina and asthma) were presented by SPs. This study was conducted in Xi'an, China, on August 17–28, 2017, and from July 30 to August 10, 2018. Eight female SPs and 2 male SPs in 2017 and 7 female SPs and a male SP in 2018 were recruited from local communities. It should be emphasized that 6 of these SPs (5 female SPs and a male SP) in 2017 also participated in the survey of 2018. Therefore, the total number of SPs recruited was 12. The SP procedure was in case development, script and checklist, SP recruitment and training, investigator training and SP exit survey, and SP visits could be found in Appendix 1.

Xi'an is the capital of Shaanxi Province, located in northwest China, with a population of 8.8 million in an area of over 9,983 km^2^; 73.4% of residents in Xi'an lived in urban areas in 2018. The per-capita gross regional product (GRP) was US$12,103 in 2018. We selected all urban districts of Xi'an as our samples. There were 65 CHCs in the selected areas, and all of them were invited to participate; however, two CHCs declined because they provided only public health, not basic medical health. In total, this study consisted of 63 CHCs.

### Physician Survey

Physician survey was conducted 3 months prior to the standardized patient visit to collect information on physicians at each CHC, including demographic characteristics, working experience, education, and qualification. In view of the busy work of physicians, and in order not to add additional burden for the physicians, the questionnaire is left for physicians to fill in and return.

### Measurement of PCC

We evaluated PCC on the three dimensions based on the framework in Figure 1. The history taking, physical and laboratory examination, and feeling were used to measure exploring disease and illness experience (PCC1). Specifically, the number of recommended (essential) questions asked and recommended (essential) exams performed in the clinical checklists were used to measure disease experience. For recommended and essential items, we gave weights for 2 and 1, respectively. Using a 5-point Likert scale (e.g., strongly agree, agree, uncertain, disagree, strongly disagree), one question (the physician made you feel free that you were willing to show your symptoms and fears) was captured to measure illness experience (feeling), ranging from 5 to 1. The total score of PCC1 was ranging from 0 to 64. Family address, family history, and employment were used to measure understanding the whole person (PCC2), ranging from 0 to 3. Five questions were used to measure finding the common ground (PCC3), including (1) you agreed with the physician's opinion about the problem; (2) the physician fully explained this problem to you; (3) the physician fully explained the treatment plan to you; (4) whether the physician advised discussing your roles in disease management; (5) overall, do you like this physician? The first three questions were measured using a 5-point Likert scale. The total score of PCC3 was ranging from 0 to 17. More details of the scoring methods can be found in Appendix 2.

### Evaluating the Quality of Primary Care

We measured the quality of the primary care by (1) accuracy of diagnosis, (2) consultation time, (3) appropriateness of treatment, (4) unnecessary exams, (5) unnecessary drugs, and (6) medical expenditure. The SPs were instructed to directly ask physicians at the end of the visit if a diagnosis was initially offered. The diagnosis was classified as “correct” if the physicians gave any one of the correct diagnoses according to the pre-determined criteria. For unstable angina, the correct diagnoses included unstable angina, angina, or coronary heart disease; for asthma, the correct diagnoses included asthma or allergic asthma. Diagnose time means the time length physicians spent with SPs for consultation, and it was regarded as a proxy for provider effort (31). The treatment was defined as correct if the provider prescribed any one of the correct medications. Referrals to tertiary hospitals or secondary hospitals were also a correct treatment for unstable angina according to WHO guidelines (32). Unstable angina is an intermediate state between acute myocardial infarction and stable angina. Due to its unique pathophysiological mechanism and clinical characteristics, if it is not properly and timely treated, the patient may develop acute myocardial infarction. To avoid delays in inpatient visits due to the low quality of the primary care facilities (e.g., the lack of inspection equipment and qualified physicians), a referral was also recorded as the correct treatment for unstable angina. An unnecessary exam and/or drug was defined as the exams and/or drug that was prescribed by the physicians was unnecessary or even harmful for the case-specific SP. We included all necessary exams and drugs in our predesigned checklist and thus the item was regarded as unnecessary if it did not fall into the checklist. All examinations and drugs were listed and determined by a panel of professors, doctors, and pharmacists as correct/essential, palliative, and unnecessary or harmful (34). Medical expenditure mainly included visit fees, exam expenses, and drug expenses in the primary medical setting. For each visit, SPs paid a visit fee and purchased all prescribed medications. We calculated all expense of exams physicians performed and planned to perform although SPs received all non-invasive tests and rejected all invasive tests. The clinical checklist, and criteria of correct diagnoses, and treatment could be found in Table 1.

**Table 1:** Evaluation criteria for Unstable Angina and Asthma.

	**Unstable Angina**	**Asthma**
Clinical checklist	•Blood pressure •Pulse •Auscultation •Temperature Electrocardiogram	Auscultation •Blood pressure •Pulse •Temperature
Accuracy of diagnosis	1. Correct diagnosis Unstable angina, angina, or coronary heart disease 2. Incorrect diagnosis (provided by primary care providers) Myocardial ischemia, neuralgia, menopausal syndrome, myocarditis, cervical spondylosis, chest congestion, swelling in chest	1. Correct diagnosis Asthma, or allergic asthma 2.Incorrect diagnosis (provided by primary care providers) Heart disease, cold, coronary heart disease, respiratory infection, myocardial ischemia, acute pharyngitis, mycoplasma infection
Appropriateness of treatment	1. Correct treatment Aspirin, clopidogrel/ or other anti-platelet agents, referral. 2. Unnecessary or harmful (provided by providers) Antibiotics, ginkgo oral liquid, Naoxintong capsule, oral electrolyte solution, psychiatric medication, theophylline	1. Correct treatment Bronchodilators, theophylline, inhaled or oral corticosteroids, leukotriene inhibitors, inhaled anticholinergic 2. Unnecessary or harmful (provided by providers) Aspirin, compound liquorice tablet, erythromycin, lung-nourishing semifluid extract, ginkgo oral liquid, cephalosporin

### Statistical Analysis

Our analysis unit was the interaction between physicians and standardized patients. Ordinary least-squares regression models with fixed effects were used for the continuous variables and logistic regression models with fixed effects were used for the categorical variables. Our econometric specification is:


(1) $$\begin{array}{r} y_{ijt}=\beta_{0}+\beta_{1}PCC_{ijt}+\beta_{2}X_{ijt}+\beta_{3}W_{ijt}+\pi_{ij}+\delta_{ij}+\varphi_{t}+\mu_{ijt}\\ \;+v_{ijt}+\varepsilon_{ijt} \end{array}$$

where *y*_*ijt*_ represents the quality of the primary care indicator that was analyzed in the community health center i district j on day t. PCC represents the total score of patient-centered communication. *X*_*ijt*_ is a set of the observable demographic correlates of the physicians (gender and age) and the SPs (gender). *W*_*ijt*_ is a set of the observable character of community health centers (CHC Character and health alliance). π_*ij*_ indicates community health center fixed effects, δ_*ij*_ indicates district fixed effects and μ_*ijt*_ indicates disease fixed effects. φ_*t*_ indicates year fixed effects. *v*_*ijt*_ indicates SPs fixed effects. ε_*ijt*_ is the error term. Robust standard errors were clustered at the community health center level. All analyses were performed using Stata (vision 15, Stata Corp LP, College Station, Texas, USA).

### Sensitivity Analysis

We examined the consistency of the results regarding the following: (1) controlling for different potential confounding factors (i.e., with and without missing data in the regression models); (2) using two different cases; (3) using two different years; and (4) normalizing measurement of PCC sub-domains.

### Ethics

The ethics approval was obtained by the Ethics Committee of Xi'an Jiaotong University Health Science Center (approval number: 2015-406). We are approved to record the interactions between physicians and standardized patients using a concealed recording device. Written consents were obtained from the physician and the director of each CHCs along with a face-to-face survey ~3 months before standardized patient visits. Additionally, written consent was obtained from each SP.

## Results

### Basic Characteristics

This study included 492 interactions across two cases. Among them, interactions from public CHCs accounted for 414 out of 492 (84.15%), and interactions from CHCs within health alliance accounted for 436 out of 492 (88.62%). Also, 54.47% of physicians were female, and 36.79% of them aged between 40 and 50 years old. Most SPs were female (83.54%). The average working experience of physicians was 22.87 years. Most were practicing (assistant) physician (95.82%), and had an educational level at junior college and above (59.83%). More details can be found in Table 2.

**Table 2:** Characteristics of interactions between physicians and SPs.

	**Case**				**Year**				**Overall**	
	**Unstable angina**		**Asthma**		**Year 2017**		**Year 2018**			
	**No**.	**%**	**No**.	**%**	**No**.	**%**	**No**.	**%**	**No**.	**%**
**CHC character**										
Public	207	84.49	207	83.81	210	84.68	204	83.61	414	84.15
Private	38	15.51	40	16.19	38	15.32	40	16.39	78	15.85
*N*	245		247		248		244		492	
**Health alliance**										
Yes	217	88.57	219	88.66	220	88.71	216	88.52	436	88.62
No	28	11.43	28	11.34	28	11.29	28	11.48	56	11.38
*N*	245		247		248		244		492	
**SP gender**										
Female	219	89.39	192	77.73	197	79.44	214	87.70	411	83.54
Male	26	10.61	55	22.27	51	20.56	30	12.30	81	16.46
*N*	245		247		248		244		492	
**Physician age group**										
Age <30	16	6.53	12	4.86	9	3.63	19	7.79	28	5.69
30 ≤ Age <40	72	29.39	50	20.24	59	23.79	63	25.82	122	24.80
40 ≤ Age <50	86	35.10	95	38.46	97	39.11	84	34.43	181	36.79
Age >= 50	71	28.98	90	36.44	83	33.47	78	31.97	161	32.72
*N*	245		247		248		244		492	
**Physician gender**										
Female	128	52.24	140	56.68	119	47.98	149	61.07	268	54.47
Male	117	47.76	107	43.32	129	52.02	95	38.93	224	45.53
*N*	245		247		248		244		492	
Physician working experience (years), mean, S.D.	22.83	12.58	22.90	11.68	23.58	12.56	21.73	11.29	22.87	12.09
*N*	114		125		147		92		239	
**Physician education**										
Technical secondary school and blow	49	42.98	47	37.60	64	43.54	32	34.78	96	40.17
Junior college and above	65	57.02	78	62.40	83	54.46	60	65.22	143	59.83
*N*	114		125		147		92		239	
**Practicing (assistant) physician**										
Yes	109	95.61	120	96.00	141	95.92	88	95.65	229	95.82
No	5	4.39	5	4.00	6	4.08	4	4.35	10	4.18
*N*	114		125		147				239	

*N refers to the number of the interactions between physicians and SPs; for variables like CHC character, health alliance, SP gender, physician age, there is no missing data, thus, there are 492 interactions totally; for variables like physician working experience, physician education, and practicing (assistant) physician, they have missing data, thus, there are 239 interactions except the missing data*.

*Source: the author's calculation*.

### Scores of PCC and the Quality of Primary Care

**Figure 2:**
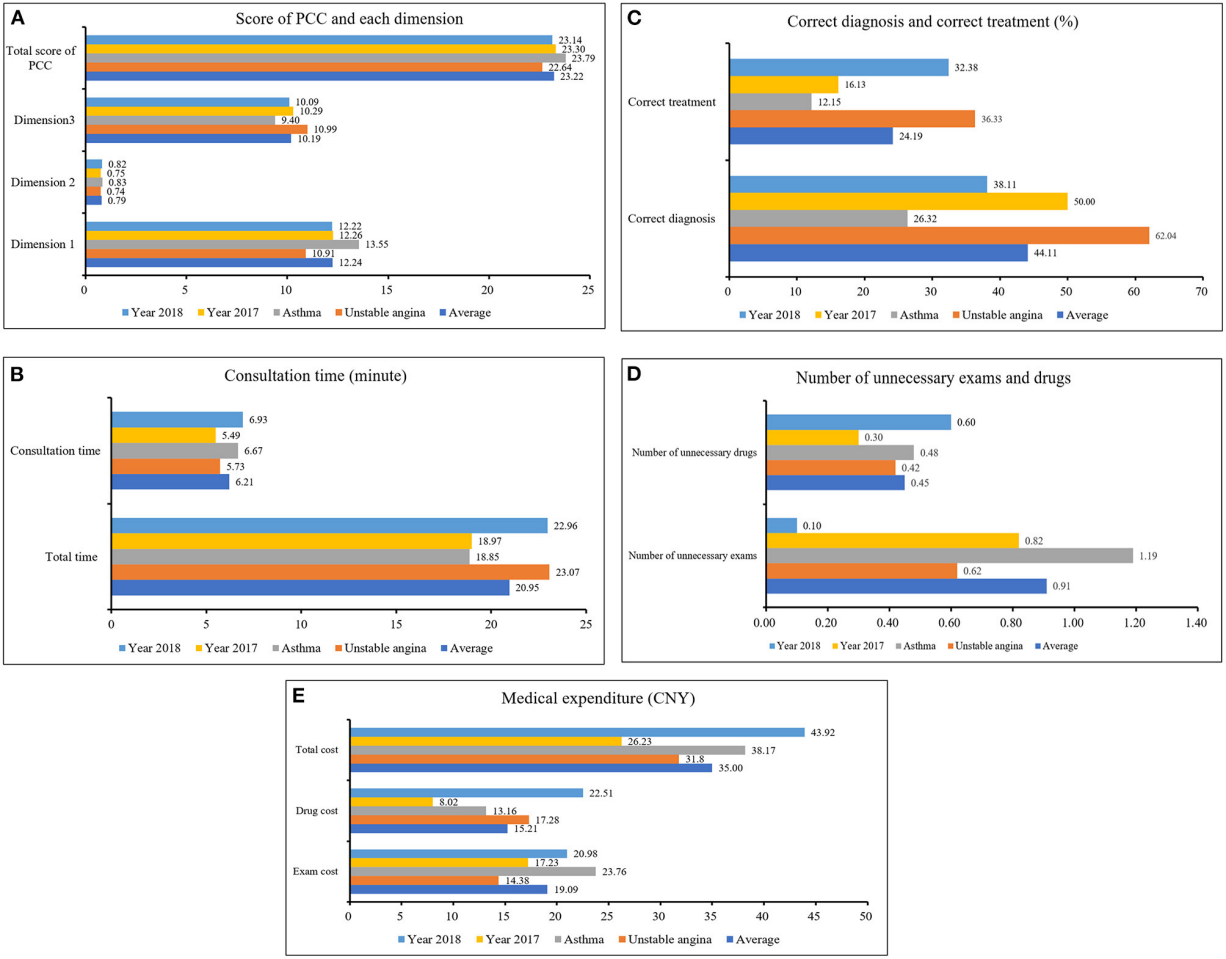
Total score of PCC and the quality of primary care. **(A)** showed the average score of PCC1, PCC2, and PCC3 was 12.24 ± 4.04 (out of 64), 0.79 ± 0.64 (out of 3), and 10.19 ± 3.60 (out of 17), respectively. The average total score of PCC was 23.22 ± 6.24 (out of 84). Source: the author's calculation. **(B)** showed the average total consultation time and diagnosis time was 20.95 ± 11.70 min, and 6.21 ± 4.52 min, respectively. Source: the author's calculation. **(C)** From the frequency of correct diagnosis and correct treatment of primary care in CHCs in urban China, we found 44.11% of the visits (217 out of 492) having correct diagnosis, and 24.19% of the visits (119 out of 492) having correct treatment. Source: the author's calculation. **(D)** showed the number of unnecessary exams and drugs was 0.91 ± 1.05 items, and 0.45 ± 0.82 items, respectively. Source: the author's calculation. **(E)** showed the average exam cost, drug cost and total cost was 19.09 ± 29.04 CNY, 15.21 ± 29.26 CNY, and 35.00 ± 41.26 CNY, respectively. Source: the author's calculation.

Figure 2 showed the average score of PCC1, PCC2, and PCC3 was 12.24 ± 4.04 (out of 64), 0.79 ± 0.64 (out of 3), and 10.19 ± 3.60 (out of 17), respectively. The average total score of PCC was 23.22 ± 6.24 (out of 84). The average total consultation time and diagnosis time was 20.95 ± 11.70 min, and 6.21 ± 4.52 min, respectively. We found 44.11% of the visits (217 out of 492) having a correct diagnosis, and 24.19% of the visits (119 out of 492) having correct treatment. The average number of unnecessary exams and drugs was 0.91 ± 1.05, and 0.45 ± 0.82, respectively. The average total cost was 35.00 ± 41.26 CNY, respectively.

### Comparing PCC and Primary Care Quality Across Basic Characteristics

In Table 3, we found that CHC character (public or private) would affect the score of PCC1 (*P* < 0.10). Health alliance would affect the score of f PCC1 (*P* < 0.10), and the score of PCC2 (*P* < 0.01). Physician's age would affect the score of PCC1 (*P* < 0.05). Physician's working experience would affect the total score of PCC (*P* < 0.01), the score of the PCC1 (*P* < 0.05), PCC2 (*P* < 0.10), and PCC3 (*P* < 0.05). Practicing (assistant) physician qualification would affect the score of PCC1 (*P* < 0.10).

**Table 3:** Comparing PCC and the quality of the primary care across basic characteristics.

	**PCC Mean (S.D.)**	**PCC1 Mean (S.D.)**	**PCC2 Mean (S.D.)**	**PCC3 Mean (S.D.)**	**Correct diagnosis (%)**	**Correct treatment (%)**	**Consultation time Mean (S.D.)**	**Number of unnecessary exams Mean (S.D.)**	**Number of unnecessary drugs Mean (S.D.)**	**Medical expenditure (CNY) Mean (S.D.)**
**CHC Character**										
Public	23.15 (6.18)	12.09 (4.05)	0.78 (0.64)	10.28 (3.62)	43.24	25.60	6.12 (4.27)	0.86 (1.00)	0.47 (0.84)	34.31 (40.39)
Private	23.55 (6.55)	13.00 (3.95)	0.82 (0.62)	9.73 (3.49)	48.72	16.67	6.65 (5.67)	1.18 (1.26)	0.31 (0.69)	38.67 (45.76)
*P*-value	0.607	0.070	0.629	0.219	0.371	0.091	0.440	0.035	0.603	0.393
**Health alliance**										
Yes	23.37 (6.33)	12.35 (4.10)	0.82 (0.64)	10.20 (3.64)	43.35	23.17	6.39 (4.64)	0.90 (1.06)	0.44 (0.79)	36.40 (42.13)
No	22.02 (5.40)	11.38 (3.50)	0.55 (0.51)	10.09 (3.30)	50.00	32.14	4.80 (3.14)	0.98 (1.00)	0.48 (1.06)	24.07 (32.07)
*P*-value	0.127	0.089	0.001	0.823	0.345	0.140	0.001	0.578	0.789	0.011
**Physician age group**										
Age <30	23.54 (6.78)	12.07 (4.15)	0.64 (0.62)	10.82 (4.02)	53.57	42.86	6.86 (4.19)	0.57 (0.79)	0.43 (0.63)	17.74 (23.99)
30 ≤ Age <40	23.87 (6.13)	12.35 (4.15)	0.77 (0.63)	10.75 (3.43)	51.64	30.33	6.02 (4.04)	0.96 (1.17)	0.39 (0.69)	34.19 (43.32)
40 ≤ Age <50	22.97 (6.33)	12.31 (4.43)	0.75 (0.62)	9.91 (3.54)	45.30	22.10	5.96 (4.37)	0.94 (1.04)	0.43 (0.85)	33.80 (38.38)
Age ≥ 50	22.95 (6.14)	12.10 (3.68)	0.88 (0.65)	9.98 (3.71)	35.40	18.63	6.51 (5.06)	0.89 (1.00)	0.52 (0.91)	39.96 (44.42)
*P*-value	0.891	0.020	0.958	0.650	0.031	0.012	0.051	0.057	0.003	0.001
**Physician gender**										
Female	23.32 (6.45)	12.45 (4.15)	0.78 (0.62)	10.09 (3.58)	45.15	26.49	6.37 (4.66)	0.97 (1.05)	0.42 (0.80)	35.91 (43.68)
Male	23.09 (5.98)	11.99 (3.91)	0.79 (0.66)	10.31 (3.63)	42.86	21.43	6.01 (4.35)	0.83 (1.05)	0.48 (0.85)	33.91 (38.24)
*P*-value	0.677	0.208	0.848	0.511	0.610	0.191	0.391	0.155	0.389	0.591
**Physician working experience (years)**										
<20	24.90 (6.87)	13.09 (6.78)	4.15 (0.95)	10.87 (3.77)	49.46	25.81	6.34 (4.02)	0.95 (1.11)	0.41 (0.76)	24.06 (32.20)
≥20	22.55 (6.20)	11.92 (6.20)	4.08 (0.77)	9.86 (3.75)	42.47	21.92	6.50 (5.20)	0.99 (1.12)	0.46 (0.82)	43.00 (47.80)
*P*-value	0.006	0.033	0.059	0.044	0.289	0.489	0.784	0.751	0.635	<0.001
**Physician education**										
Middle school and blow	23.02 (6.19)	12.11 (4.11)	0.84 (0.51)	10.06 (3.88)	43.75	20.83	6.22 (4.43)	1.02 (1.15)	0.43 (0.79)	37.93 (43.09)
High school and above	23.77 (6.74)	12.55 (4.16)	0.83 (0.73)	10.38 (3.73)	46.15	25.17	6.58 (4.99)	0.94 (1.09)	0.45 (0.80)	34.08 (43.59)
*P*-value	0.386	0.431	0.954	0.520	0.714	0.437	0.564	0.602	0.846	0.502
**Practicing (assistant) physician**										
Yes	26.40 (6.85)	14.70 (3.23)	0.90 (0.57)	10.80 (4.57)	50.00	10.00	9.78 (6.58)	0.80 (0.79)	0.80 (0.79)	22.36 (22.19)
No	23.34 (6.49)	12.27 (4.15)	0.84 (0.65)	10.23 (3.75)	44.98	24.02	6.29 (4.63)	0.98 (1.12)	0.42 (0.79)	36.21 (43.97)
*P*-value	0.147	0.069	0.769	0.643	0.755	0.306	0.023	0.612	0.144	0.093
**Cases**										
Unstable angina	22.64 (6.02)	10.91 (3.76)	0.74 (0.61)	10.99 (3.34)	62.04	36.33	5.73 (4.81)	0.62 (1.04)	0.42 (0.65)	31.80 (38.49)
Asthma	23.79 (6.41)	13.55 (3.89)	0.83 (0.66)	9.40 (3.69)	26.32	12.15	6.67 (4.17)	1.19 (0.99)	0.48 (0.96)	38.17 (43.69)
*P*-value	0.043	<0.001	0.112	<0.001	<0.001	<0.001	0.021	<0.001	0.407	0.087
**Year**										
2017	23.30 (6.54)	12.26 (4.33)	0.75 (0.70)	10.29 (3.75)	50.00	16.13	5.49 (4.11)	0.82 (0.98)	0.30 (0.74)	26.23 (33.96)
2018	23.14 (5.93)	12.22 (3.74)	0.82 (0.56)	10.09 (3.45)	38.11	32.38	6.93 (4.81)	1.00 (1.12)	0.60 (0.87)	43.92 (45.94)
*P*-value	0.772	0.911	0.223	0.555	0.008	<0.001	<0.001	0.068	<0.001	<0.001

*Chi-2 test was used for dummy variable; Univariate ANOVAS was employed for continuous variable. Source: the author's calculation*.

We found that CHC character (public or private) would affect the number of unnecessary exams (*P* < 0.05). Health alliance would affect consultation time (*P* < 0.01), and medical expenditure (*P* < 0.05). Physician's age would affect the correct diagnosis (*P* < 0.05) and correct treatment (*P* < 0.05), consultation time (*P* < 0.10), the number of unnecessary exams (*P* < 0.10) and unnecessary drugs (*P* < 0.01), and medical expenditure (*P* < 0.01). Physician's working experience would affect medical expenditure (*P* < 0.001). Practicing (assistant) physician qualification would affect consultation time (*P* < 0.05), and medical expenditure (*P* < 0.10). More details could be found in Table 3.

### Association Between PCC and the Quality of Primary Care

Table 3 (and Table 2 in Appendix 3) showed the results of the correlation between the total score of PCC and the quality of primary care. After controlling for the potential confounding factors and fixed effects, the PCC increased the correct diagnosis by 10 percentage points (*P* < 0.01), the correct treatment by 7 percentage points (*P* < 0.01), the consultation time by 0.17 min (*P* < 0.01), the number of unnecessary drugs by 0.03 items (*P* < 0.01), and the medical expenditure by 1.46 CNY (*P* < 0.01). Specifically, interactions with a higher score of PCC1 were more likely to have a correct diagnosis (increased by 7 percentage points, *P* < 0.05), more consultation time (increased by 0.32 min, *P* < 0.05), more unnecessary exams (increased by 0.03 items, *P* < 0.10), more unnecessary drugs (increased by 0.03 items, *P* < 0.01), and higher medical expenditure (increased by 2.17 CNY, *P* < 0.01). The PCC2 increased the consultation time by 1.60 min (*P* < 0.01), the number of unnecessary drugs by 0.26 items (*P* < 0.01), and medical expenditure by 7.54 CNY (*P* < 0.05). Interactions with a higher score of PCC3 were more likely to have a correct diagnosis (increased by 24 percentage points, *P* < 0.01), correct treatment (increased by 19 percentage points, *P* < 0.01), more consultation time (increased by 0.13 min, *P* < 0.05), more unnecessary drugs (increased by 0.06 items, *P* < 0.01), and higher medical expenditure (increased by 1.85 CNY, *P* < 0.01) (Table 4).

**Table 4:** Association between patient-centered communication and primary care quality.

	**Correct diagnosis**				**Correct treatment**				**Medical expenditure**			
	**(1)**	**(2)**	**(3)**	**(4)**	**(5)**	**(6)**	**(7)**	**(8)**	**(9)**	**(10)**	**(11)**	**(12)**
	**Coef. (S.E.)**	**Coef. (S.E.)**	**Coef. (S.E.)**	**Coef. (S.E.)**	**Coef. (S.E.)**	**Coef. (S.E.)**	**Coef. (S.E.)**	**Coef. (S.E.)**	**Coef. (S.E.)**	**Coef. (S.E.)**	**Coef. (S.E.)**	**Coef. (S.E.)**
**PCC**	0.10^***^ (0.02)				0.07^***^ (0.02)				1.46^***^ (0.28)			
**PCC1**		0.07^**^ (0.03)				0.03 (0.04)				2.17^***^ (0.48)		
**PCC2**			0.12 (0.17)				0.10 (0.25)				7.54^**^ (3.01)	
**PCC3**				0.24^***^ (0.04)				0.19^***^ (0.04)				1.85^***^ (0.53)
Private	0.44 (0.35)	0.40 (0.34)	0.44 (0.34)	0.66^*^ (0.34)	−0.60^**^ (0.30)	−0.56^**^ (0.28)	−0.52^*^ (0.27)	−0.49^*^ (0.28)	4.81 (6.50)	3.61 (6.45)	5.18 (6.74)	6.00 (6.62)
Non-alliance	0.30^***^ (0.45)	0.11^**^ (0.47)	0.03^**^ (0.46)	0.10^***^ (0.41)	0.69 (0.50)	0.54 (0.44)	0.53 (0.44)	0.62 (0.56)	−3.53 (11.83)	−3.22 (11.39)	−2.46 (11.56)	−6.41 (11.94)
SP gender	0.91^***^ (0.33)	0.89^***^ (0.30)	0.85^***^ (0.30)	0.90^**^ (0.35)	0.46 (0.29)	0.53^*^ (0.30)	0.52^*^ (0.30)	0.28 (0.31)	5.97 (4.10)	6.99^*^ (4.16)	6.49 (4.40)	5.37 (4.27)
30–39	−0.13 (0.27)	−0.08 (0.26)	−0.09 (0.26)	−0.21 (0.28)	−0.16 (0.25)	−0.14 (0.25)	−0.14 (0.25)	−0.23 (0.25)	−3.70 (3.99)	−3.11 (4.02)	−3.62 (4.08)	−4.14 (4.08)
40–49	−0.46 (0.56)	−0.34 (0.51)	−0.32 (0.49)	−0.45 (0.56)	−0.82^*^ (0.43)	−0.69^*^ (0.41)	−0.70^*^ (0.40)	−0.84^*^ (0.43)	20.58^***^ (6.89)	21.00^***^ (6.81)	20.53^***^ (7.16)	21.35^***^ (7.44)
≥50	−0.66 (0.57)	−0.54 (0.51)	−0.52 (0.49)	−0.60 (0.56)	−0.96^**^ (0.44)	−0.88^**^ (0.41)	−0.89^**^ (0.41)	−0.95^**^ (0.45)	20.25^***^ (6.89)	20.07^***^ (6.70)	18.92^***^ (6.87)	20.90^***^ (7.34)
Physician gender	−0.96^*^ (0.58)	−0.86 (0.52)	−0.89^*^ (0.51)	−1.02^*^ (0.57)	−0.88^*^ (0.49)	−0.83^*^ (0.48)	−0.86^*^ (0.48)	−0.88^*^ (0.49)	24.08^***^ (7.92)	24.56^***^ (7.72)	21.82^***^ (7.86)	23.82^***^ (8.49)
Year	0.64 (1.15)	0.32 (0.17)	−0.01 (1.09)	0.39 (1.05)	1.43 (1.41)	1.10 (1.32)	0.96 (1.32)	1.46 (1.43)	9.76 (14.69)	10.97 (15.31)	−1.16 (16.29)	3.45 (14.94)
Case	−0.65^***^ (0.26)	−0.72^***^ (0.24)	−0.42^***^ (0.22)	−0.53^***^ (0.24)	−0.68^***^ (0.31)	−0.67^***^ (0.32)	−0.59^***^ (0.32)	−0.32^***^ (0.33)	3.79 (3.94)	−0.26 (3.83)	4.96 (3.90)	8.44^**^ (4.09)
*N*	492	492	492	492	492	492	492	492	492	492	492	492
*R* ^2^	0.20	0.16	0.16	0.24	0.19	0.17	0.16	0.21	0.25	0.24	0.22	0.23

*Standard errors in second column;*

* *p < 0.1*,

** *p <0.05*,

*** *p < 0.01; (1) ~ (12) represents 12 different models; Ordinary least-squares regression models with fixed effects were used for the continuous variables (Medical expenditure) and logistic regression models with fixed effects were used for the categorical variables (Correct diagnosis, and Correct treatment). Source: the author's calculation*.

### Sensitivity Analysis Outcomes

Tables 3, 4 in Appendix 4 showed the sensitivity analysis results for unstable angina. The results were close to our original analysis results. For example, unstable angina interactions with a higher total score of PCC were more likely to have a correct diagnosis (increased by 16 percentage points, *P* < 0.01), more consultation time (increased by 0.16 min, *P* < 0.01), more unnecessary exams (increased by 0.03 items, *P* < 0.05), more unnecessary drugs (increased by 0.02 items, *P* < 0.05), and higher medical expenditure (increased by 1.11 CNY, *P* < 0.01). Furthermore, the correlations between each dimension of PCC and the quality of primary care for unstable angina were also analyzed. Specifically, interactions with a higher score of PCC1 were more likely to have a correct diagnosis (increased by 9 percentage points, *P* < 0.05), more consultation time (increased by 0.34 min, *P* < 0.01), more unnecessary exams (increased by 0.05 items, *P* < 0.5), and higher medical expenditure (increased by 1.31 CNY, *P* < 0.1). Interactions with a higher score of PCC1 were less likely to have a correct treatment (decreased by 9 percentage points, *P* < 0.10). The PCC2 increased the correct diagnosis by 47 percentage points (*P* < 0.10), the consultation time by 1.31 min (*P* < 0.01), the number of unnecessary drugs by 0.21 items (*P* < 0.01), and medical expenditure by 10.28 CNY (*P* < 0.05). Interactions with a higher score of PCC2 were less likely to have a correct treatment (decreased by 61 percentage points, *P* < 0.10). Interactions with a higher score of PCC3 were more likely to have a correct diagnosis (increased by 42 percentage points, *P* < 0.01), correct treatment (increased by 11 percentage points, *P* < 0.10), more unnecessary drugs (increased by 0.04 items, *P* < 0.01), and higher medical expenditure (increased by 1.48 CNY, *P* < 0.1). We also conducted the sensitivity analysis for asthma. The results were close to our original analysis results. More detailed results of sensitivity analysis could be found in Appendix 4.

## Discussion

To our knowledge, this study is the first to evaluate the status quo of PCC and its association with the quality of primary care using standardized patient approach and internationally developed framework of PCC in the context of China. This study revealed poor communication quality between primary care providers and patients in primary care settings in urban China. Further, our findings extend observational studies that showed the PCC model in the primary care settings has positive associations with the quality of the primary care. Interactions with a higher score of PCC were more likely to have a correct diagnosis and correct treatment, and more consultation time, more unnecessary drugs, and higher medical expenditure. Additionally, this study has significant practical implications for realizing the Healthy China initiative. In October 2016, the Chinese government announced the Healthy China 2030 blueprint, which requires marked improvement in the quality and level of the health services, and reinforces the role of primary care. Primary care could play in screening and monitoring for COVID-19, and maintaining routine care on other health conditions (9, 35). The effectiveness of these directives depends on whether we could build a strong primary care system with high quality (9, 35). The findings of this study could be helpful to identify the challenges of patient-physician communication in primary care settings. This also could be helpful to develop intervention strategies to promote patient-centered communication and the quality of primary care. In terms of the representativeness of the sample, in a national program, there were 308 public CHCs (82.13%) and 67 private CHCs (17.87%). It could be seen that the breakdown of public CHCs and private CHCs in our study reflected ratio of the two models of care in the whole area, and in urban China more broadly (36). Additionally, according to the Health Statistics Yearbook of 2020, most physicians of CHCs aged between 25 and 54 years old in year of 2018 (31.1% aged between 25 and 34 years old, 32.2% aged between 35 and 44 years old, and 22.1% aged between 45 and 54 years old). The ratio of those who have worked for 10–19 years, 20–29 years, and 30 years and above was 26.5, 24.3, and 15.2 (37). All these characteristics have demonstrated the representativeness of our sample.

Previous studies have shown that there was no gold standard approach to measure PCC (38). In this study, PCC was measured using standardized patient approach. SP approach, which focuses on direct communications between physicians and patients and measures the quality according to the standardized clinical checklist, minimizes recall bias, and subjective bias. A previous study conducted in Canada used 4 subjective items to measure patient's perceptions that their illness experience has been explored using a 4-point Likert scale, such as “to what extent was your main problem discussed today” (16). This method is arbitrary and mainly focuses on the patient's perceptions. Another study conducted in the United States measured PCC1 by the extent of exploration by physicians (39). In that study, the physician's responses were divided into 5 items: ignore patient's statement (cutoff), ask one question (preliminary exploration), ask two or more questions (further exploration), and express empathy or understanding the reason for this visit. This method mainly focuses on the number of questions preformed in the interaction between physician and patient, rather than the quality of these questions. A previous study conducted in China observed physicians' verbal communication and behaviors using a self-developed subjective assessment instrument. The physicians' performance was evaluated using a 5-point Likert scale from “terrible performance” (1 point) to “perfect performance” (5 points) (7). We combined both quantity and quality of the questions and exams (recommended and essential items) performed by the physicians according to the standardized clinical checklists, as well as the patient's feeling. Our study focused on direct communications between physicians and patients in real primary care settings by recording, minimizes recall bias, and subjective bias.

The current study revealed poor communication between primary care providers and patients, as well as the poor quality of primary care. There could be various explanations. The first possible explanation might be related to the problems in the health system. For example, the physician has a dominant position in physician-patient communication in the context of China. They are much less attentive to the patient's feelings, expectations, needs, and discouraging patients from asking questions and presenting themselves. A previous study showed that almost 38% of physicians from provincial hospitals took only 4 min for each outpatient (40). In our study, physicians from CHCs spent 6 min on average for each visit in primary settings. Our study also showed nearly 30% of patients were interrupted during the consultations. Physicians feel they do not have enough time to listen, explain, and discuss with their patients (40). In case, patients were not able to fully describe their symptoms, and express their initial concerns. In such a physician-centered and disease-centered model, it was impossible to “understand the whole person” and facilitate shared decision-making (40). The second reason was the lack of medical training. Our study indicated that almost 40% of physicians had an educational level at technical secondary school and blow, which was highly related to the insufficient financial incentives in primary care. It was well-documented the pay for primary care physicians was much lower than the average income in China and two times lower than the majority of OECD countries (41). The financial incentives were also scarce and 40% of primary care physicians had no pension in China (41). Without sufficient financial incentives, physicians' work satisfaction was also weakened and the prevalence of intention to quit the job was high, accounting for 56% in CHCs (41). Also, only less skilled physicians might select themselves into CHCs (42–44). A previous study has revealed 3,775 (36%) of 10,626 primary care physicians participated in no training program in 2015 (41). Furthermore, training on patient-centered communication skills and the relationship between physicians and patients received only scant attention in our health system (7).

The most important finding of the study indicated significantly positive associations between PCC and correct diagnosis, correct treatment, consultation time, the number of unnecessary drugs, and higher medical expenditure. Das et al. proposed a two-stage (consultation stage, and treatment stage) model of provider-patient interaction (34). We followed this model to interpret our results. In the consultation stage, a physician's main task is to identify the true situation of the patient based on their initial symptoms, and their expectation and feelings. The physician exerts costly effort to explore the true situation according to the number of the clinical checklist items performed, communication time, and examinations even though sometimes it's clinically unnecessary. In the treatment stage, the physician provides the treatments based on the belief in the consultation stage. Das et al. proposed that the choice of the treatment is determined by the physician's desire to cure the patient (which is facilitating by a more accurate diagnosis), as well as the market incentives for overtreatment (34). In the PCC model, a physician could pay more attention to elicit and understand patient perspectives (e.g., problems, concerns, ideas, expectations, needs, and feelings). Also, a patient may be more active in expressing their worries, concerns, thoughts, and participating in decision-making on treatment opinions, which all could improve doctor-patient relationships, improve patient satisfaction and trust, and thus improve health outcomes (19). As a result, the rate of correct diagnosis and correct treatment improved in our study. Our study also showed negative associations between PCC and the number of unnecessary drugs, and medical expenditure. This may be because in practice, physicians will choose efforts and treatments to maximize their own benefits (e.g., curing their patients, overall health, and financial rewards) (31). That is why a physician dispended more numbers of unnecessary drugs in the interactions. In order to determine the source of the associations, the correlations between each dimension of PCC and the quality of primary care were also analyzed. The results showed that the influence mainly derived from PCC3 (namely communication skills) and PCC1 (namely clinical capacity), which could provide path choice to improve PCC and thus the quality of primary care.

This study should be interpreted in the context of several potential limitations. Firstly, the standardized patient approach is limited to cases that are easier to be portrayed without obvious physical symptoms and with low-risk invasive examinations. It's worth noting that the severity of the two cases is quite different which can have an effect on the physician-patient communication. Thus, the outcomes based on these diseases may not be representative of the broader diseases treated by primary care physicians. Secondly, our study only concentrated on correlation analysis. Finally, because of the missing data related to physicians' sociodemographic characteristics, and the characteristics of facilities, more pieces of evidence are needed in the next research steps. Bearing the limitations in mind, this study has highlighted several strategies that might be helpful to improve the PCC. First, the clinical capacity of primary care providers needs to be strengthened. Second, the methods to improve and assess communication skills should be expanded. For example, courses and training that discuss current deficiencies in physician-patient communication, reasons for the deficiency, adverse consequences for physician and patient, and specific communication skills are essential for primary care providers (45). Furthermore, physicians should have the opportunity to practice their skills and to receive feedback about their performance by using a standardized patient approach. Also, communication skills should be included in the quality assessment system. Third, the crucial role of the physician-patient relationship in altering physicians' behaviors must be fully understood. Actions must be taken to formulate the PCC model and rebuild a healthy mode of interaction. Finally, strategies on reforming the pay structure to better reflect the value of physicians and to provide a stronger motivation for performance improvement are urgently needed (19).

## Conclusion

This study revealed poor communication between primary care providers and patients, as well as the poor quality of primary care. The PCC model has not been achieved, which could be one source of the intensified physician-patient relationship. Our findings showed the PCC model in the primary care settings has positive associations with the quality of the primary care. Interactions with a higher score of PCC were more likely to have a correct diagnosis and correct treatment, and more consultation time, more unnecessary drugs, and higher medical expenditure. To improve PCC, the clinical capacity and communication skills of primary care providers need to be strengthened. Also, strategies on reforming the pay structure to better reflect the value of physicians and to provide a stronger motivation for performance improvement are urgently needed. The findings of the study could lead to the development of strategies to improve the quality of patient-physician communication and the primary care quality in urban China. It is anticipated that our findings will inform policymakers in China.

## Data Availability Statement

The raw data supporting the conclusions of this article will be made available by the authors, without undue reservation.

## Ethics Statement

The studies involving human participants were reviewed and approved by Ethics Committee of Xi'an Jiaotong University Health Science Center (No. 2015-406). The patients/participants provided their written informed consent to participate in this study.

## Author Contributions

MS: conception and design, data analysis and interpretation, writing-original draft, and supervision. ZZ: conception and design, funding acquisition, and supervision. YS and XF review and editing. All authors read and approved the final manuscript.

## Funding

This work was supported by China Medical Board (grant number 15-277), National Natural Science Foundation of China (grant number 72164031), and Natural Science Foundation of Inner Mongolia (grant number 2020BS07002).

## Conflict of Interest

The authors declare that the research was conducted in the absence of any commercial or financial relationships that could be construed as a potential conflict of interest.

## Publisher's Note

All claims expressed in this article are solely those of the authors and do not necessarily represent those of their affiliated organizations, or those of the publisher, the editors and the reviewers. Any product that may be evaluated in this article, or claim that may be made by its manufacturer, is not guaranteed or endorsed by the publisher.
