# Supplementary material for: The Association Between Patient-Centered Communication and Primary Care Quality in Urban China: Evidence From a Standardized Patient Study
Source: Front Public Health. 2022 Feb 4;9:779293. doi: 10.3389/fpubh.2021.779293 (PMC8854212; doi:10.3389/fpubh.2021.779293)
Supplement: Supplementary file 1 [file Table_1.DOCX]

Supplementary Material

**Appendix 1: Standardized Patient procedure**

# Case selection and scenario

An SP is a healthy individual recruited from the local community and trained to portray an actual patient’s historical, physical and emotional features in a standardized way in a real setting. In our study, SPs were trained to portray unstable angina and asthma. Unstable angina and asthma were chosen because 1) these diseases had a high incidence in China (approximately 8% and 2%, respectively, for people aged 50 and older); 2) these diseases are easier to portray without obvious symptoms and with a low risk of invasive examinations; and 3) these diseases were selected from SP cases employed in previous international studies and those conducted in China. Two scenarios were considered in this study: For unstable angina, a 50-year-old patient had chest pain recently, and it felt like there was something heavy pressing on the chest. For asthma, a 40-year-old patient had a problem with breathing, and last night, it became terrible.

# Script and checklist

Physicians and professors with rich experience in conducting SP studies were invited to develop our scripts. These scripts were based on those used in two previous studies in India and rural China, and were adapted for use in urban China by the research team, which included consulting physicians from both tertiary hospitals and community health centres, and professors with rich experience in conducting SP studies (Das et al. 2012; Sylvia et al. 2015). The scripts covered all possible questions a physician may ask and examinations they might perform during the interaction. Each script included 1) a detailed background story for each case; 2) an opening statement that highlighted the symptoms to be portrayed by the SPs; 3) an illness history presented in question-and-answer format; and 4) a list of possible examinations and treatments. The checklists of specific cases provided a list of questions that a physician should ask and examinations that should be performed during the interactions.

# Recruitment and training of standardized patient

SPs were recruited from local communities to ensure that they were representative of actual patients commonly diagnosed by primary care providers. The SPs were chosen following these basic criteria: 1) SPs must be in good physical condition without confounding symptoms; 2) non-physicians were recruited as SPs because physicians’ knowledge and behaviors may impact diagnosis and treatment; 3) a reasonable level of intelligence and memory and communication ability were essential; and 4) SPs should match the re-designed cases in terms of age.

The SPs participated in a three-day training conducted by a team consisting of professors, medical experts, and investigators. The training included the following: 1) the details of case scenarios and scripts were explained to the SPs; 2) the recordings of interactions between SPs and physicians obtained from our pilot study were presented to the SPs; 3) role-playing and one-on-one training were used to help SPs understand and memorize the scripts and portray the cases; 4) some principles and scripts for responding to the physician’s questions were provided to help the SPs avoid examinations; and 5) an assessment of the SPs’ performance.

# Standardized patient visit

We were approved to record the interactions between physicians and SPs using a concealed recording device. Written consent was obtained from the physician and the director of each CHC, and a face-to-face survey was completed approximately 3 months prior to SP visits.

Four SPs were randomly assigned to and independently visited each CHC (two SPs portraying unstable angina and two SPs portraying asthma). The SPs could not choose the physicians, and they must be seen by whoever would have seen them had they been a common patient once they entered the practical setting. The SPs wore a concealed recording device to record the interactions between physicians and the SPs.

# Investigator training and standardized patient exit survey

Ten postgraduate students attended 3 workshops to train as investigators to complete the exit surveys of the SPs immediately after their visits based on the visit recordings. Three methods were used to collect data on the SP-physician interactions. First, the SPs wore a concealed recording device that allowed us to accurately rate the interactions without recall bias. Second, the SPs participated in a case-specific ‘debriefing survey’ upon exiting the CHCs. This survey covered the interactions as well as the SP’s impressions of the providers. Finally, the SPs purchased all medications prescribed and paid all fees so that information on the medications dispensed and fees charged could be collected.

**Appendix 2: Scoring methods of patient-centered communication**

Table S1 Scoring methods of patient-centered communication

| Patient-centered communication | | Scoring |
| --- | --- | --- |
| **1.** **Exploring disease and illness experience** | | |
| **1.1 Exploring disease** **experience** | | |
| **1.1.1** | Number of recommended questions asked | 1 if physician asked the recommended question, 0 otherwise. Calculate the total score of all the recommended items. |
| **1.1.2** | Number of recommended exams performed | 1 if physician preformed the recommended exam, 0 otherwise. Calculate the total score of all the recommended items. |
| **1.1.3** | Number of essential questions asked | 2 if physician asked the essential question, 0 otherwise. Calculate the total score of all the essential items. |
| **1.1.4** | Number of essential exams performed | 2 if physician preformed the essential exam, 0 otherwise. Calculate the total score of all the essential items. |
| **1.2 Exploring illness experience** | | |
| **1.2.1** | The physician made you feel free that you were willing to show your symptoms and fears | Using 5-point Likert scale (e.g. strongly agree, agree, uncertain, disagree, strongly disagree); ranging from 5 to 1. |
| **2. Understanding the whole person** | |  |
| **2.1** | Area of family | 1 if physician asked address of family, 0 otherwise. |
| **2.2** | Family history | 1 if physician asked family history, 0 otherwise. |
| **2.3** | Employment | 1 if physician asked employment, 0 otherwise. |
| **3. Finding the common ground** | | |
| **3.1** | You agreed with the physician’s opinion about the problem | Using 5-point Likert scale (e.g. strongly agree, agree, uncertain, disagree, strongly disagree); ranging from 5 to 1. |
| **3.2** | The physician fully explained this problem to you | Using 5-point Likert scale (e.g. strongly agree, agree, uncertain, disagree, strongly disagree); ranging from 5 to 1. |
| **3.3** | The physician fully explained the treatment plan to you | Using 5-point Likert scale (e.g. strongly agree, agree, uncertain, disagree, strongly disagree); ranging from 5 to 1. |
| **3.4** | Whether the physician gave advice to discuss your roles in disease management | 1 if physician gave advice to discuss patient’s roles in disease management, 0 otherwise. |
| **3.5** | Overall, do you like this physician | 1 if patient likes this physician, 0 otherwise. |

**Appendix 3: Association between PCC and the quality of primary care**

Table S2 Association between patient-centered communication and primary care quality

|  | Consultation time | | | | Number of unnecessary exams | | | | Number of unnecessary drugs | | | |
| --- | --- | --- | --- | --- | --- | --- | --- | --- | --- | --- | --- | --- |
|  | (1) | （2） | （3） | (4) | (5) | （6） | （7） | (8) | (9) | （10） | （11） | (12) |
|  | Coef.  (S.E.) | Coef.  (S.E.) | Coef.  (S.E.) | Coef.  (S.E.) | Coef.  (S.E.) | Coef.  (S.E.) | Coef.  (S.E.) | Coef.  (S.E.) | Coef.  (S.E.) | Coef.  (S.E.) | Coef.  (S.E.) | Coef.  (S.E.) |
| **PCC** | 0.17^***^  (0.03) |  |  |  | 0.01 |  |  |  | 0.03^***^  (0.01) |  |  |  |
| **PCC1** |  | 0.32^***^  (0.06) |  |  |  | 0.03^*^  (0.01) |  |  |  | 0.03^***^  (0.01) |  |  |
| **PCC2** |  |  | 1.60^***^  (0.34) |  |  |  | 0.05  (0.09) |  |  |  | 0.26^***^  (0.08) |  |
| **PCC3** |  |  |  | 0.13^**^  (0.06) |  |  |  | -0.01  (0.02) |  |  |  | 0.06^***^  (0.01) |
| Private | 0.19  (0.63) | 0.01  (0.60) | 0.24  (0.63) | 0.28  (0.65) | 0.35^**^  (0.01) | 0.33^**^  (0.15) | 0.35^**^  (0.14) | 0.35^**^  (0.14) | -0.14  (0.11) | -0.16  (0.11) | -0.14  (0.10) | -0.11  (0.10) |
| Non-alliance | -1.57^***^  (0.58) | -1.44^**^  (0.64) | -1.07^*^  (0.62) | -1.90^***^  (0.63) | -0.01  (0.19) | 0.02  (0.18) | 0.01  (0.20) | -0.01  (0.21) | 0.36^*^  (0.18) | 0.34^*^  (0.17) | 0.42^**^  (0.17) | 0.29  (0.18) |
| SP gender | 0.09  (0.46) | 0.23  (0.45) | 0.16  (0.48) | 0.07  (0.48) | 0.35^**^  (0.15) | 0.36^**^  (0.15) | 0.35^**^  (0.15) | 0.36^**^  (0.15) | -0.23^**^  (0.11) | -0.21^*^  (0.11) | -0.22^*^  (0.11) | -0.26^**^  (0.11) |
| 30-39 | -0.42  (0.44) | -0.34  (0.45) | -0.41  (0.44) | -0.45  (0.45) | -0.09  (0.11) | -0.08  (0.11) | -0.09  (0.11) | -0.08  (0.11) | 0.02  (0.08) | 0.03  (0.08) | 0.03  (0.08) | 0.01  (0.08) |
| 40-49 | -0.95  (1.10) | -0.92  (1.07) | -1.08  (0.99) | -0.83  (1.10) | 0.35^*^  (0.18) | 0.35^*^  (0.18) | 0.35^*^  (0.15) | 0.36^*^  (0.19) | 0.01  (0.13) | 0.03  (0.15) | -0.01  (0.13) | 0.02  (0.14) |
| ≥50 | -0.86  (1.07) | -0.89  (1.05) | -1.16  (0.95) | -0.80  (1.07) | 0.29  (0.18) | 0.29  (0.18) | 0.28  (0.18) | 0.29  (0.19) | 0.02  (0.13) | 0.02  (0.15) | -0.03  (0.13) | 0.04  (0.14) |
| Physician gender | -0.69  (1.09) | -0.61  (1.05) | -1.13  (0.95) | -0.73  (1.10) | 0.22  (0.16) | 0.23  (0.18) | 0.21  (0.19) | 0.22  (0.19) | 0.09  (0.13) | 0.09  (0.15) | 0.02  (0.14) | 0.08  (0.14) |
| Year | 1.56  (1.34) | 2.02  (1.34) | 0.15  (1.45) | 0.66  (1.48) | 0.39  (0.48) | 0.48  (0.48) | 0.34  (0.48) | 0.33  (0.47) | 0.02  (0.26) | -0.05  (0.27) | -0.25  (0.32) | -0.10  (0.27) |
| Case | 0.70  (0.42) | 0.05  (0.47) | 0.77^*^  (0.41) | 1.10^***^  (0.39) | 0.53^***^  (0.09) | 0.47^***^  (0.10) | 0.53^***^  (0.09) | 0.52^***^  (0.09) | 0.05  (0.07) | 0.01  (0.08) | 0.07  (0.07) | 0.19^**^  (0.08) |
| *N* | 492 | 492 | 492 | 492 | 492 | 492 | 492 | 492 | 492 | 492 | 492 | 492 |
| *R*^2^ | 0.26 | 0.27 | 0.26 | 0.22 | 0.17 | 0.19 | 0.19 | 0.19 | 0.18 | 0.14 | 0.16 | 0.19 |

Note: Standard errors in second column; ^*^ *p* < .1, ^**^ *p* < .05, ^***^ *p* < .01; (1) ~ (12) represents 12 different models; Ordinary least-squares regression models with fixed effects were used for the continuous variables (e.g., Consultation time, Number of unnecessary exams, Number of unnecessary drugs).

**Appendix 4: *Sensitivity analysis outcomes***

Table S7 and Table S8 in appendix 4 showed the sensitivity analysis results for the year 2017. The results were close to our original analysis results. For example, interactions in the year 2017 with a higher total score of PCC were more likely to have a correct diagnosis (increased by 13 percentage points, P<0.01), correct treatment (increased by 6 percentage points, P<0.05), more consultation time (increased by 0.16 minutes, P<0.01), more unnecessary drugs (increased by 0.03 items, P<0.01), and higher medical expenditure (increased by 1.59 CNY, P<0.01). Furthermore, the correlations between each dimension of PCC and the quality of primary care in the year 2017 were also analyzed. Specifically, interactions with a higher score of PCC1 were more likely to have a correct diagnosis (increased by 8 percentage points, P<0.10), more consultation time (increased by 0.25 minutes, P<0.01), more unnecessary drugs (increased by 0.04 items, P<0.01), and higher medical expenditure (increased by 2.59 CNY, P<0.10). The PCC2 increased the consultation time by 1.69 minutes (P<0.1), and the number of unnecessary drugs by 0.19 items (P<0.05). Interactions with a higher score of PCC3 were more likely to have a correct diagnosis (increased by 29 percentage points, P<0.01), correct treatment (increased by 21 percentage points, P<0.01), more consultation time (increased by 0.13 minutes, P<0.05), more unnecessary drugs (increased by 0.05 items, P<0.01), and higher medical expenditure (increased by 1.77 CNY, P<0.01). We also conducted the sensitivity analysis in the year 2018.

- Table S7, Table S8, Table S9 and Table S10 in appendix 4 around here-

Table S11 in appendix 4 showed the sensitivity analysis results controlling for different potential confounding factors. In the primary analysis, we did not control physician working experience, education, and practicing (assistant) physician because of the missing data; in this sensitivity analysis, these variables were controlled. The results were close to our original analysis results. For example, interactions with a higher total score of PCC were more likely to have a correct diagnosis (increased by 14 percentage points, P<0.01), correct treatment (increased by 11 percentage points, P<0.05), more consultation time (increased by 0.09 minutes, P<0.10), more unnecessary drugs (increased by 0.04 items, P<0.01), and higher medical expenditure (increased by 1.64 CNY, P<0.01). Furthermore, the correlations between each dimension of PCC and the quality of primary care were also analyzed. Specifically, interactions with a higher score of PCC1 were more likely to have a correct diagnosis (increased by 10 percentage points, P<0.05), more consultation time (increased by 0.16 minutes, P<0.05), and higher medical expenditure (increased by 2.60 CNY, P<0.01). The PCC2 increased the consultation time by 0.74 minutes (P<0.10), the number of unnecessary drugs by 0.34 items (P<0.01), and the medical expenditure by 7.11 CNY (P<0.05). Interactions with a higher score of PCC3 were more likely to have a correct diagnosis (increased by 30 percentage points, P<0.01), correct treatment (increased by 21 percentage points, P<0.01), more unnecessary drugs (increased by 0.07 items, P<0.01), and higher medical expenditure (increased by 1.91 CNY, P<0.05).

- Table S11 in appendix 4 around here-

Table S12 in appendix 4 showed the sensitivity analysis results for the normalization results for the score of PCC, PCC1, PCC2, and PCC3. The results were close to our original analysis results. For example, interactions with a higher total score of PCC were more likely to have a correct diagnosis (increased by 65 percentage points, P<0.01), correct treatment (increased by 47 percentage points, P<0.01), more consultation time (increased by 1.07 minutes, P<0.01), more unnecessary drugs (increased by 0.21 items, P<0.01), and higher medical expenditure (increased by 9.10 CNY, P<0.01). Furthermore, the correlations between each dimension of PCC and the quality of primary care were also analyzed. Specifically, interactions with a higher score of PCC1 were more likely to have a correct diagnosis (increased by 27 percentage points, P<0.05), more consultation time (increased by 1.29 minutes, P<0.01), more unnecessary exams (increased by 0.11 items, P<0.10), more unnecessary drugs (increased by 0.13 items, P<0.01), and higher medical expenditure (increased by 8.77 CNY, P<0.1). The PCC2 increased the consultation time by 1.01 minutes (P<0.01), the number of unnecessary drugs by 0.16 items (P<0.01), the medical expenditure by 4.79 CNY (P<0.05). Interactions with a higher score of PCC3 were more likely to have a correct diagnosis (increased by 88 percentage points, P<0.01), correct treatment (increased by 68 percentage points, P<0.01), more consultation time (increased by 0.45 minutes, P<0.05), more unnecessary drugs (increased by 0.22 items, P<0.01), and higher medical expenditure (increased by 4.79 CNY, P<0.05).

- Table S12 in appendix 4 around here-

Table S3 Association between patient-centered communication and primary care quality for Unstable Angina

|  | Correct diagnosis | | | | Correct treatment | | | | Medical expenditure | | | |
| --- | --- | --- | --- | --- | --- | --- | --- | --- | --- | --- | --- | --- |
|  | (1) | （2） | （3） | (4) | (5) | （6） | （7） | (8) | (9) | （10） | （11） | (12) |
|  | Coef.  (S.E.) | Coef.  (S.E.) | Coef.  (S.E.) | Coef.  (S.E.) | Coef.  (S.E.) | Coef.  (S.E.) | Coef.  (S.E.) | Coef.  (S.E.) | Coef.  (S.E.) | Coef.  (S.E.) | Coef.  (S.E.) | Coef.  (S.E.) |
| **PCC** | 0.16^***^  (0.04) |  |  |  | -0.01  (0.03) |  |  |  | 1.11^***^  (0.41) |  |  |  |
| **PCC1** |  | 0.09^**^  (0.04) |  |  |  | -0.09^*^  (0.05) |  |  |  | 1.31^*^  (0.67) |  |  |
| **PCC2** |  |  | 0.47^*^  (0.27) |  |  |  | -0.61^*^  (0.34) |  |  |  | 10.28^**^  (4.36) |  |
| **PCC3** |  |  |  | 0.42^***^  (0.09) |  |  |  | 0.11^*^  (0.06) |  |  |  | 1.48^*^  (0.74) |
| Private | 0.23  (0.45) | 0.26  (0.44) | 0.41  (0.43) | 0.71^*^  (0.43) | -0.50  (0.44) | -0.35  (0.47) | -0.47  (0.43) | -0.52  (0.42) | -2.68  (6.46) | -3.58  (6.52) | -1.62  (6.84) | -0.35  (6.72) |
| Non-alliance | 0.24  (0.51) | -0.04  (0.63) | -0.07  (0.59) | 0.04  (0.38) | 0.68  (0.63) | 0.68  (0.64) | 0.54  (0.62) | 0.78  (0.65) | 1.03  (10.93) | -0.54  (10.11) | 1.13  (11.46) | -0.47  (11.43) |
| SP gender | 0.11  (0.81) | 0.61  (0.75) | 0.36  (0.72) | -1.31  (1.02) | 0.10  (0.78) | -0.06  (0.79) | 0.13  (0.81) | -0.57  (0.92) | 1.21  (13.19) | 6.66  (12.87) | 2.98  (12.84) | -2.01  (13.70) |
| 30-39 | 0.17  (0.35) | 0.32  (0.33) | 0.30  (0.31) | 0.04  (0.37) | 0.38  (0.38) | 0.40  (0.38) | 0.38  (0.37) | 0.32  (0.37) | 0.17  (4.83) | 1.04  (4.92) | 1.14  (4.73) | 0.36  (4.75) |
| 40-49 | 0.20  (0.61) | 0.04  (0.54) | -0.09  (0.53) | 0.28  (0.59) | -0.37^**^  (0.68) | -0.45^**^  (0.71) | -0.28^*^  (0.74) | -0.37^**^  (0.69) | 13.75  (10.55) | 13.08  (10.62) | 11.19  (10.31) | 13.84  (10.98) |
| ≥50 | -0.46  (0.59) | -0.54  (0.54) | -0.61  (0.55) | -0.41  (0.62) | -0.48^**^  (0.73) | -0.56^**^  (0.76) | -0.46^*^  (0.78) | -0.48^**^  (0.76) | 10.41  (10.45) | 9.61  (10.53) | 8.38  (10.41) | 10.11  (10.94) |
| Physician gender | -0.83  (0.64) | -0.17^*^  (0.61) | -0.20^**^  (0.60) | -0.73  (0.66) | -0.04^**^  (0.91) | -0.16^**^  (0.95) | -0.99^**^  (0.98) | -0.01^**^  (0.90) | 11.05  (11.43) | 9.33  (11.15) | 6.91  (10.64) | 10.30  (12.00) |
| Year | 0.05  (0.50) | 0.10  (0.53) | -0.47  (0.52) | -0.89  (0.47) | 0.85  (0.81) | 0.64  (0.77) | 0.12^*^  (0.89) | 0.86  (0.92) | 1.51  (16.82) | 3.33  (18.24) | -5.52  (17.16) | -2.52  (16.77) |
| *N* | 245 | 245 | 245 | 245 | 245 | 245 | 245 | 245 | 245 | 245 | 245 | 245 |
| *R*^2^ | 0.18 | 0.12 | 0.11 | 0.23 | 0.21 | 0.22 | 0.22 | 0.22 | 0.32 | 0.31 | 0.32 | 0.31 |

Note: Standard errors in second column; ^*^ *p* < .1, ^**^ *p* < .05, ^***^ *p* < .01; (1) ~ (12) represents 12 different models.

Table S4 Association between patient-centered communication and primary care quality for Unstable Angina

|  | Consultation time | | | | Number of unnecessary exams | | | | Number of unnecessary drugs | | | |
| --- | --- | --- | --- | --- | --- | --- | --- | --- | --- | --- | --- | --- |
|  | (1) | （2） | （3） | (4) | (5) | （6） | （7） | (8) | (9) | （10） | （11） | (12) |
|  | Coef.  (S.E.) | Coef.  (S.E.) | Coef.  (S.E.) | Coef.  (S.E.) | Coef.  (S.E.) | Coef.  (S.E.) | Coef.  (S.E.) | Coef.  (S.E.) | Coef.  (S.E.) | Coef.  (S.E.) | Coef.  (S.E.) | Coef.  (S.E.) |
| **PCC** | 0.16^***^  (0.06) |  |  |  | 0.03^**^  (0.01) |  |  |  | 0.02^**^  (0.01) |  |  |  |
| **PCC1** |  | 0.34^***^  (0.09) |  |  |  | 0.05^**^  (0.02) |  |  |  | 0.01  (0.01) |  |  |
| **PCC2** |  |  | 1.31^***^  (0.48) |  |  |  | 0.14  (0.11) |  |  |  | 0.21^***^  (0.07) |  |
| **PCC3** |  |  |  | -0.01  (0.09) |  |  |  | 0.03  (0.02) |  |  |  | 0.04^***^  (0.01) |
| Private | 0.12  (0.98) | -0.30  (0.98) | 0.28  (0.96) | 0.34  (0.97) | 0.34^*^  (0.19) | 0.29  (0.19) | 0.37^**^  (0.17) | 0.39^**^  (0.18) | -0.23^**^  (0.10) | -0.22^**^  (0.10) | -0.21^**^  (0.10) | -0.18^*^  (0.10) |
| Non-alliance | 1.51  (1.42) | 1.50  (1.34) | 1.47  (1.60) | 1.01  (1.35) | 0.26  (0.31) | 0.23  (0.31) | 0.21  (0.32) | 0.20  (0.34) | 0.09  (0.15) | 0.05  (0.16) | 0.10  (0.14) | 0.09  (0.17) |
| SP gender | -3.08^**^  (1.28) | -2.05^*^  (1.13) | -2.79^**^  (1.24) | -2.56^*^  (1.40) | -0.13  (0.31) | 0.03  (0.31) | -0.06  (0.31) | -0.16  (0.31) | 0.10  (0.16) | 0.17  (0.16) | 0.12  (0.16) | -0.02  (0.18) |
| 30-39 | -0.48  (0.60) | -0.42  (0.60) | -0.34  (0.60) | -0.27  (0.61) | -0.04  (0.15) | -0.02  (0.15) | -0.01  (0.15) | -0.02  (0.16) | 0.08  (0.09) | 0.10  (0.09) | 0.10  (0.08) | 0.07  (0.09) |
| 40-49 | -1.45  (1.40) | -1.50  (1.37) | -1.80  (1.35) | -1.62  (1.49) | 0.17  (0.22) | 0.16  (0.21) | 0.13  (0.20) | 0.17  (0.21) | 0.04  (0.15) | 0.03  (0.17) | -0.01  (0.14) | 0.06  (0.16) |
| ≥50 | -2.18  (1.37) | -2.20^*^  (1.32) | -2.46^*^  (1.28) | -2.41^*^  (1.42) | -0.14  (0.23) | -0.15  (0.22) | -0.19  (0.22) | -0.16  (0.23) | -0.02  (0.17) | -0.04  (0.19) | -0.06  (0.16) | -0.01  (0.17) |
| Physician gender | -1.86  (1.33) | -1.91  (1.26) | -2.45^*^  (1.24) | -2.38^*^  (1.38) | 0.01  (0.27) | -0.03  (0.26) | -0.10  (0.25) | -0.04  (0.27) | 0.02  (0.16) | -0.03  (0.18) | -0.06  (0.15) | 0.03  (0.17) |
| Year | 2.09  (2.35) | 2.93  (2.32) | 1.14  (2.68) | 1.63  (2.60) | 0.03  (0.67) | 0.13  (0.67) | -0.10  (0.66) | -0.07  (0.67) | -0.08  (0.29) | -0.09  (0.31) | -0.21  (0.30) | -0.16  (0.28) |
| *N* | 245 | 245 | 245 | 245 | 245 | 245 | 245 | 245 | 245 | 245 | 245 | 245 |
| *R*^2^ | 0.39 | 0.42 | 0.39 | 0.36 | 0.19 | 0.20 | 0.18 | 0.18 | 0.38 | 0.36 | 0.39 | 0.39 |

Note: Standard errors in second column; ^*^ *p* < .1, ^**^ *p* < .05, ^***^ *p* < .01; (1) ~ (12) represents 12 different models; Ordinary least-squares regression models with fixed effects were used for the continuous variables (e.g., Consultation time, Number of unnecessary exams, Number of unnecessary drugs).

Table S5 Association between patient-centered communication and primary care quality for Asthma

|  | Correct diagnosis | | | | Correct treatment | | | | Medical expenditure | | | |
| --- | --- | --- | --- | --- | --- | --- | --- | --- | --- | --- | --- | --- |
|  | (1) | （2） | （3） | (4) | (5) | （6） | （7） | (8) | (9) | （10） | （11） | (12) |
|  | Coef.  (S.E.) | Coef.  (S.E.) | Coef.  (S.E.) | Coef.  (S.E.) | Coef.  (S.E.) | Coef.  (S.E.) | Coef.  (S.E.) | Coef.  (S.E.) | Coef.  (S.E.) | Coef.  (S.E.) | Coef.  (S.E.) | Coef.  (S.E.) |
| **PCC** | 0.11^***^  (0.04) |  |  |  | 0.27^***^  (0.05) |  |  |  | 1.84^***^  (0.40) |  |  |  |
| **PCC1** |  | 0.08  (0.05) |  |  |  | 0.23^***^  (0.07) |  |  |  | 2.96^***^  (0.65) |  |  |
| **PCC2** |  |  | -0.30  (0.27) |  |  |  | 1.02^*^  (0.53) |  |  |  | 4.17  (4.50) |  |
| **PCC3** |  |  |  | 0.33^***^  (0.08) |  |  |  | 0.56^***^  (0.09) |  |  |  | 2.75^***^  (0.83) |
| Private | 0.82^*^  (0.46) | 0.71^*^  (0.43) | 0.65  (0.44) | 0.95^*^  (0.52) | -0.94  (0.73) | -0.76  (0.71) | -0.89  (0.80) | -1.18^*^  (0.65) | 14.98  (9.58) | 14.57  (9.45) | 13.21  (9.60) | 14.44  (9.57) |
| Non-alliance | 0.68^***^  (0.14) | 0.34^***^  (0.08) | 0.06^***^  (0.97) | 0.56^***^  (0.02) | -0.67  (0.82) | -0.13  (0.66) | -0.76  (0.58) | -0.86  (0.75) | -13.82  (14.94) | -12.21  (15.42) | -12.31  (14.34) | -17.76  (14.42) |
| SP gender | 0.67^***^  (0.59) | 0.45^***^  (0.55) | 0.43^***^  (0.52) | 0.09^***^  (0.62) | 0.85^**^  (0.92) | 0.39^*^  (0.83) | 0.39^*^  (0.73) | 0.29^**^  (0.04) | 14.11^*^  (7.08) | 11.00  (7.07) | 12.07  (7.31) | 16.19^**^  (7.34) |
| 30-39 | -0.84^**^  (0.39) | -0.89^**^  (0.40) | -0.92^**^  (0.41) | -0.84^**^  (0.41) | -0.42  (0.61) | -0.66  (0.54) | -0.93  (0.60) | -0.39  (0.72) | -3.34  (6.65) | -4.17  (6.86) | -6.00  (7.25) | -3.81  (6.66) |
| 40-49 | -0.61^*^  (0.90) | -0.37  (0.84) | -0.18  (0.82) | -0.69^*^  (0.97) | -0.24  (0.95) | -0.09  (0.85) | 0.05  (0.91) | 0.13  (1.03) | 25.28^**^  (12.23) | 25.18^**^  (12.45) | 30.05^**^  (12.43) | 28.12^**^  (12.60) |
| ≥50 | -0.46  (0.95) | -0.26  (0.88) | -0.02  (0.85) | -0.42  (0.95) | -086  (0.25) | -0.43  (0.10) | -0.06  (0.11) | -0.31  (0.09) | 29.19^**^  (11.77) | 29.10^**^  (11.81) | 32.02^***^  (11.63) | 31.79^**^  (12.12) |
| Physician gender | -0.44  (0.98) | -0.10  (0.91) | -0.86  (0.87) | -0.78^*^  (0.03) | -0.10  (0.94) | 0.15  (0.86) | 0.18  (0.95) | -0.06  (1.00) | 33.61^***^  (12.14) | 36.11^***^  (12.10) | 37.16^***^  (12.33) | 34.43^***^  (12.41) |
| Year | 0.71  (0.75) | 0.14  (0.71) | 0.71  (0.67) | 0.95  (0.71) | 0.82^***^  (0.28) | 0.44^***^  (0.88) | 0.67^***^  (0.91) | 0.93^***^  (0.25) | 17.27  (26.29) | 17.91  (25.99) | 1.00  (26.20) | 10.47  (26.14) |
| *N* | 247 | 247 | 247 | 247 | 247 | 247 | 247 | 247 | 247 | 247 | 247 | 247 |
| *R*^2^ | 0.21 | 0.16 | 0.16 | 0.26 | 0.32 | 0.20 | 0.19 | 0.37 | 0.33 | 0.32 | 0.27 | 0.31 |

Note: Standard errors in second column; ^*^ *p* < .1, ^**^ *p* < .05, ^***^ *p* < .01; (1) ~ (12) represents 12 different models; Ordinary least-squares regression models with fixed effects were used for the continuous variables (Medical expenditure) and logistic regression models with fixed effects were used for the categorical variables (Correct diagnosis, and Correct treatment).

Table S6 Association between patient-centered communication and primary care quality for Asthma

|  | Consultation time | | | | Number of unnecessary exams | | | | Number of unnecessary drugs | | | |
| --- | --- | --- | --- | --- | --- | --- | --- | --- | --- | --- | --- | --- |
|  | (1) | （2） | （3） | (4) | (5) | （6） | （7） | (8) | (9) | （10） | （11） | (12) |
|  | Coef.  (S.E.) | Coef.  (S.E.) | Coef.  (S.E.) | Coef.  (S.E.) | Coef.  (S.E.) | Coef.  (S.E.) | Coef.  (S.E.) | Coef.  (S.E.) | Coef.  (S.E.) | Coef.  (S.E.) | Coef.  (S.E.) | Coef.  (S.E.) |
| **PCC** | 0.23^***^  (0.04) |  |  |  | -0.005  (0.01) |  |  |  | 0.04^***^  (0.01) |  |  |  |
| **PCC1** |  | 0.36^***^  (0.06) |  |  |  | 0.01  (0.02) |  |  |  | 0.05^***^  (0.12) |  |  |
| **PCC2** |  |  | 1.90^***^  (0.35) |  |  |  | -0.03  (0.11) |  |  |  | 0.23^*^  (0.12) |  |
| **PCC3** |  |  |  | 0.30^***^  (0.08) |  |  |  | -0.03  (0.02) |  |  |  | 0.09^***^  (0.02) |
| Private | 0.55  (0.62) | 0.49  (0.55) | 0.37  (0.53) | 0.46  (0.70) | 0.32  (0.20) | 0.34^*^  (0.20) | 0.33^*^  (0.19) | 0.31  (0.19) | -0.01  (0.16) | -0.03  (0.17) | -0.05  (0.16) | -0.01  (0.15) |
| Non-alliance | -4.43^***^  (1.06) | -4.24^***^  (1.09) | -3.30^***^  (1.20) | -4.89^***^  (1.04) | -0.25  (0.22) | -0.23  (0.21) | -0.26  (0.23) | -0.21  (0.22) | 0.45  (0.34) | 0.47  (0.33) | 0.58^*^  (0.29) | 0.34  (0.31) |
| SP gender | 0.27  (0.63) | -0.12  (0.64) | -0.01  (0.68) | 0.47  (0.67) | 0.44^**^  (0.22) | 0.44^**^  (0.22) | 0.45^**^  (0.22) | 0.41^*^  (0.22) | -0.03  (0.17) | -0.10  (0.17) | -0.08  (0.17) | 0.05  (0.17) |
| 30-39 | 0.36  (0.61) | 0.25  (0.62) | 0.05  (0.64) | 0.27  (0.66) | -0.10  (0.18) | -0.09  (0.18) | -0.10  (0.18) | -0.12  (0.18) | -0.03  (0.13) | -0.07  (0.14) | -0.10  (0.13) | -0.03  (0.13) |
| 40-49 | -0.57  (1.28) | -0.57  (1.28) | -0.17  (1.29) | -0.18  (1.36) | 0.66^*^  (0.36) | 0.62^*^  (0.36) | 0.65^*^  (0.35) | 0.67^*^  (0.35) | -0.01  (0.25) | 0.02  (0.27) | 0.08  (0.24) | 0.04  (0.23) |
| ≥50 | 0.11  (1.33) | 0.11  (1.34) | 0.08  (1.28) | 0.45  (1.39) | 0.89^***^  (0.32) | 0.86^***^  (0.32) | 0.89^***^  (0.32) | 0.90^***^  (0.31) | 0.04  (0.22) | 0.06  (0.24) | 0.07  (0.22) | 0.09  (0.20) |
| Physician gender | -0.83  (1.15) | -0.51  (1.14) | -0.86  (1.15) | -0.66  (1.23) | 0.62^*^  (0.34) | 0.60^*^  (0.34) | 0.62^*^  (0.34) | 0.66^**^  (0.33) | 0.18  (0.24) | 0.25  (0.26) | 0.22  (0.25) | 0.17  (0.22) |
| Year | 1.23  (1.77) | 1.25  (1.78) | -1.08  (1.74) | 0.25  (1.85) | 0.60  (0.59) | 0.70  (0.58) | 0.64  (0.57) | 0.54  (0.58) | 0.27  (0.42) | 0.17  (0.44) | -0.14  (0.49) | 0.17  (0.43) |
| *N* | 247 | 247 | 247 | 247 | 247 | 247 | 247 | 247 | 247 | 247 | 247 | 247 |
| *R*^2^ | 0.38 | 0.36 | 0.35 | 0.33 | 0.22 | 0.22 | 0.22 | 0.22 | 0.23 | 0.19 | 0.18 | 0.23 |

Note: Standard errors in second column; ^*^ *p* < .1, ^**^ *p* < .05, ^***^ *p* < .01; (1) ~ (12) represents 12 different models; Ordinary least-squares regression models with fixed effects were used for the continuous variables (e.g., Consultation time, Number of unnecessary exams, Number of unnecessary drugs).

Table S7 Association between patient-centered communication and primary care quality in year 2017

|  | Correct diagnosis | | | | Correct treatment | | | | Medical expenditure | | | |
| --- | --- | --- | --- | --- | --- | --- | --- | --- | --- | --- | --- | --- |
|  | (1) | （2） | （3） | (4) | (5) | （6） | （7） | (8) | (9) | （10） | （11） | (12) |
|  | Coef.  (S.E.) | Coef.  (S.E.) | Coef.  (S.E.) | Coef.  (S.E.) | Coef.  (S.E.) | Coef.  (S.E.) | Coef.  (S.E.) | Coef.  (S.E.) | Coef.  (S.E.) | Coef.  (S.E.) | Coef.  (S.E.) | Coef.  (S.E.) |
| **PCC** | 0.13^***^  (0.04) |  |  |  | 0.06^**^  (0.03) |  |  |  | 1.59^***^  (0.39) |  |  |  |
| **PCC1** |  | 0.08^*^  (0.05) |  |  |  | -0.03  (0.06) |  |  |  | 2.59^***^  (0.71) |  |  |
| **PCC2** |  |  | 0.13  (0.23) |  |  |  | -0.04  (0.36) |  |  |  | 3.59  (3.40) |  |
| **PCC3** |  |  |  | 0.29^***^  (0.06) |  |  |  | 0.21^***^  (0.07) |  |  |  | 1.77^***^  (0.63) |
| Private | -0.22  (0.53) | -0.29  (0.50) | -0.22  (0.47) | 0.14  (0.54) | -0.11  (0.88) | -0.04  (0.87) | -0.08  (0.88) | 0.15  (0.81) | 4.43  (4.21) | 1.66  (4.62) | 4.23  (5.05) | 6.37  (4.90) |
| Non-alliance | 0.29^***^  (0.86) | 0.85^**^  (0.84) | 0.66^**^  (0.84) | 0.94^***^  (0.75) | 0.42  (0.81) | 0.09  (0.69) | 0.15  (0.71) | 0.41  (0.87) | 0.53  (19.23) | 1.81  (19.56) | -1.88  (18.32) | -3.85  (18.21) |
| SP gender | 0.25  (0.48) | 0.41  (0.43) | 0.37  (0.42) | 0.21  (0.49) | -0.11  (0.51) | 0.13  (0.50) | 0.13  (0.48) | -0.38  (0.64) | 2.67  (4.94) | 5.31  (4.87) | 4.60  (5.35) | 3.08  (5.10) |
| 30-39 | -0.74^**^  (0.32) | -0.59^*^  (0.32) | -0.57^*^  (0.32) | -0.86^***^  (0.32) | 0.14  (0.36) | 0.24  (0.35) | 0.22  (0.34) | 0.08  (0.34) | -3.25  (5.63) | -2.57  (5.44) | -1.62  (5.74) | -2.99  (5.77) |
| 40-49 | -0.22  (0.71) | -0.15  (0.73) | -0.13  (0.70) | -0.34  (0.74) | 0.46^***^  (0.89) | 0.20^***^  (0.29) | 0.19^***^  (0.49) | 0.39^***^  (0.72) | 11.75^*^  (6.30) | 11.72  (7.08) | 12.96^*^  (7.75) | 12.19  (7.39) |
| ≥50 | -0.52  (0.81) | -0.37  (0.76) | -0.39  (0.73) | -0.60  (0.83) | 0.63^***^  (0.78) | 0.29^***^  (0.97) | 0.30^***^  (0.97) | 0.60^***^  (0.82) | 11.19^*^  (6.28) | 11.49  (6.96) | 10.73  (7.59) | 11.18  (7.28) |
| Physician gender | -0.93  (0.81) | -0.86  (0.78) | -0.95  (0.75) | -1.23  (0.83) | 0.11^***^  (0.65) | 0.65^***^  (0.04) | 0.68^***^  (0.03) | 0.10^***^  (0.84) | 21.88^***^  (6.98) | 23.15^***^  (7.76) | 20.01^**^  (8.21) | 20.11^**^  (7.87) |
| Case | -0.76^***^  (0.44) | -0.30^***^  (0.34) | -0.05^***^  (0.31) | -0.42^***^  (0.36) | -0.10^**^  (0.49) | -0.65  (0.48) | -0.75^*^  (0.45) | -0.03^*^  (0.56) | 7.81^*^  (4.10) | 4.74  (4.64) | 13.74^***^  (4.49) | 14.10^***^  (4.20) |
| *N* | 248 | 248 | 248 | 248 | 248 | 248 | 248 | 248 | 248 | 248 | 248 | 248 |
| *R*^2^ | 0.26 | 0.21 | 0.20 | 0.30 | 0.14 | 0.13 | 0.13 | 0.18 | 0.24 | 0.24 | 0.17 | 0.20 |

Note: Standard errors in second column; ^*^ *p* < .1, ^**^ *p* < .05, ^***^ *p* < .01; (1) ~ (12) represents 12 different models.

Table S8 Association between patient-centered communication and primary care quality in year 2017

|  | Consultation time | | | | Number of unnecessary exams | | | | Number of unnecessary drugs | | | |
| --- | --- | --- | --- | --- | --- | --- | --- | --- | --- | --- | --- | --- |
|  | (1) | （2） | （3） | (4) | (5) | （6） | （7） | (8) | (9) | （10） | （11） | (12) |
|  | Coef.  (S.E.) | Coef.  (S.E.) | Coef.  (S.E.) | Coef.  (S.E.) | Coef.  (S.E.) | Coef.  (S.E.) | Coef.  (S.E.) | Coef.  (S.E.) | Coef.  (S.E.) | Coef.  (S.E.) | Coef.  (S.E.) | Coef.  (S.E.) |
| **PCC** | 0.16^***^  (0.04) |  |  |  | 0.01  (0.01) |  |  |  | 0.03^***^  (0.01) |  |  |  |
| **PCC1** |  | 0.25^***^  (0.06) |  |  |  | 0.02  (0.02) |  |  |  | 0.04^***^  (0.01) |  |  |
| **PCC2** |  |  | 1.69^***^  (0.29) |  |  |  | 0.11  (0.11) |  |  |  | 0.19^**^  (0.09) |  |
| **PCC3** |  |  |  | 0.13^**^  (0.07) |  |  |  | 0.01  (0.02) |  |  |  | 0.05^***^  (0.02) |
| Private | 0.41  (0.81) | 0.13  (0.84) | 0.33  (0.80) | 0.55  (0.81) | 0.28  (0.19) | 0.27  (0.19) | 0.28  (0.18) | 0.30  (0.18) | -0.14  (0.12) | -0.18  (0.13) | -0.14  (0.13) | -0.08  (0.11) |
| Non-alliance | -1.88^***^  (0.66) | -1.76^***^  (0.62) | -1.37^*^  (0.74) | -2.31^**^  (0.92) | -0.06  (0.27) | -0.06  (0.28) | -0.03  (0.28) | -0.10  (0.27) | 0.27  (0.28) | 0.26  (0.27) | 0.28  (0.26) | 0.18  (0.25) |
| SP gender | -1.84^***^  (0.63) | -1.57^**^  (0.63) | -2.03^***^  (0.65) | -1.74^***^  (0.65) | 0.52^***^  (0.16) | 0.54^***^  (0.16) | 0.51^***^  (0.17) | 0.52^***^  (0.16) | -0.09  (0.09) | -0.04  (0.09) | -0.09  (0.09) | -0.10  (0.10) |
| 30-39 | 0.15  (0.51) | 0.22  (0.52) | 0.38  (0.51) | 0.20  (0.51) | -0.10  (0.16) | -0.09  (0.16) | -0.08  (0.16) | -0.09  (0.27) | -0.04  (0.10) | -0.02  (0.10) | 0.001  (0.10) | -0.04  (0.10) |
| 40-49 | -1.08  (1.08) | -1.08  (1.07) | -0.86  (0.95) | -1.02  (1.14) | 0.52^*^  (0.28) | 0.52^*^  (0.29) | 0.53^*^  (0.27) | 0.52^*^  (0.28) | -0.25  (0.24) | -0.25  (0.27) | -0.22  (0.24) | -0.25  (0.25) |
| ≥50 | -1.31  (1.10) | -1.28  (1.10) | -1.51  (1.02) | -1.31  (1.20) | 0.41  (0.25) | 0.41  (0.25) | 0.40^*^  (0.23) | 0.41  (0.25) | -0.27  (0.23) | -0.27  (0.27) | -0.30  (0.22) | -0.27  (0.24) |
| Physician gender | -1.43  (1.08) | -1.31  (1.10) | -1.67^*^  (0.93) | -1.61  (1.19) | 0.29  (0.24) | 0.29  (0.24) | 0.27  (0.23) | 0.27  (0.24) | -0.16  (0.25) | -0.15  (0.28) | -0.21  (0.25) | -0.20  (0.26) |
| Case | -0.30  (0.52) | -0.58  (0.54) | 0.01  (0.50) | 0.34  (0.48) | 0.50^***^  (0.13) | 0.50^***^  (0.14) | 0.53^***^  (0.12) | 0.55^***^  (0.12) | 0.20^**^  (0.08) | 0.18^**^  (0.09) | 0.29^***^  (0.09) | 0.32^***^  (0.09) |
| *N* | 248 | 248 | 248 | 248 | 248 | 248 | 248 | 248 | 248 | 248 | 248 | 248 |
| *R*^2^ | 0.25 | 0.24 | 0.27 | 0.21 | 0.21 | 0.21 | 0.22 | 0.21 | 0.23 | 0.20 | 0.19 | 0.21 |

Note: Standard errors in second column; ^*^ *p* < .1, ^**^ *p* < .05, ^***^ *p* < .01; (1) ~ (12) represents 12 different models; Ordinary least-squares regression models with fixed effects were used for the continuous variables (e.g., Consultation time, Number of unnecessary exams, Number of unnecessary drugs).

Table S9 Association between patient-centered communication and primary care quality in year 2018

|  | Correct diagnosis | | | | Correct treatment | | | | Medical expenditure | | | |
| --- | --- | --- | --- | --- | --- | --- | --- | --- | --- | --- | --- | --- |
|  | (1) | （2） | （3） | (4) | (5) | （6） | （7） | (8) | (9) | （10） | （11） | (12) |
|  | Coef.  (S.E.) | Coef.  (S.E.) | Coef.  (S.E.) | Coef.  (S.E.) | Coef.  (S.E.) | Coef.  (S.E.) | Coef.  (S.E.) | Coef.  (S.E.) | Coef.  (S.E.) | Coef.  (S.E.) | Coef.  (S.E.) | Coef.  (S.E.) |
| **PCC** | 0.12^***^  (0.04) |  |  |  | 0.09^**^  (0.04) |  |  |  | 1.33^***^  (0.50) |  |  |  |
| **PCC1** |  | 0.11^**^  (0.05) |  |  |  | 0.11^*^  (0.06) |  |  |  | 1.91^**^  (0.84) |  |  |
| **PCC2** |  |  | 0.30  (0.29) |  |  |  | 0.29  (0.23) |  |  |  | 13.30^**^  (5.64) |  |
| **PCC3** |  |  |  | 0.32^***^  (0.07) |  |  |  | 0.18^***^  (0.06) |  |  |  | 1.91^*^  (1.07) |
| Private | 0.95  (0.59) | 0.90^*^  (0.55) | 0.98^*^  (0.54) | 0.16^**^  (0.58) | -0.20^**^  (0.52) | -0.18^**^  (0.50) | -0.26^**^  (0.50) | -0.06^**^  (0.53) | 4.10  (10.32) | 3.82  (10.25) | 5.63  (10.87) | 4.81  (10.05) |
| Non-alliance | 0.91^**^  (0.43) | 0.97^**^  (0.41) | 0.92^***^  (0.36) | 0.69^*^  (0.38) | 1.32  (0.87) | 1.32  (0.86) | 1.25  (0.83) | 1.15  (0.87) | -12.85  (9.87) | -12.01  (9.63) | -8.61  (10.33) | -15.28  (10.75) |
| SP gender | 0.38^**^  (0.67) | 0.38^**^  (0.66) | 0.50^**^  (0.66) | 0.39^**^  (0.66) | 0.07^**^  (0.83) | 0.09^***^  (0.81) | 0.13^***^  (0.80) | 0.99^**^  (0.83) | 2.71  (14.11) | 3.24  (13.82) | 8.13  (14.56) | 3.02  (14.85) |
| 30-39 | 0.39  (0.41) | 0.38  (0.42) | 0.33  (0.42) | 0.39  (0.41) | -0.47  (0.38) | -0.43  (0.37) | -0.47  (0.37) | -0.49  (0.39) | -2.75  (6.59) | -2.34  (6.60) | -4.16  (6.72) | -3.28  (6.77) |
| 40-49 | -0.42  (0.62) | -0.40  (0.54) | -0.37  (0.54) | -0.12  (0.65) | -0.49^***^  (0.54) | -0.42^***^  (0.54) | -0.41^***^  (0.54) | -0.34^**^  (0.54) | 28.21^***^  (9.58) | 27.49^***^  (9.53) | 25.41^***^  (9.49) | 29.64^***^  (10.15) |
| ≥50 | -0.69  (0.65) | -0.69  (0.56) | -0.64  (0.55) | -0.43  (0.64) | -0.14^***^  (0.64) | -0.08^***^  (0.62) | -0.96^***^  (0.60) | -0.99^***^  (0.66) | 33.44^***^  (10.27) | 32.47^***^  (10.29) | 31.47^***^  (9.99) | 34.45^***^  (10.56) |
| Physician gender | -0.04  (0.66) | -0.97^*^  (0.58) | -0.52^*^  (0.59) | -0.96  (0.67) | -0.66^***^  (0.64) | -0.55^**^  (0.62) | -0.55^**^  (0.62) | -0.57^**^  (0.64) | 25.96^**^  (11.99) | 25.66^**^  (11.90) | 22.02^*^  (11.65) | 26.70^**^  (12.60) |
| Case | -0.61^***^  (0.36) | -0.93^***^  (0.39) | -0.73^***^  (0.33) | -0.85^**^  (0.36) | -0.91^***^  (0.57) | -0.20^***^  (0.58) | -0.93^***^  (0.56) | -0.41^***^  (0.61) | -1.01  (6.62) | -6.56  (6.33) | -3.95  (6.18) | 2.89  (8.15) |
| *N* | 244 | 244 | 244 | 244 | 244 | 244 | 244 | 244 | 244 | 244 | 244 | 244 |
| *R*^2^ | 0.21 | 0.18 | 0.16 | 0.25 | 0.28 | 0.27 | 0.26 | 0.28 | 0.26 | 0.26 | 0.26 | 0.25 |

Note: Standard errors in second column; ^*^ *p* < .1, ^**^ *p* < .05, ^***^ *p* < .01; (1) ~ (12) represents 12 different models; Ordinary least-squares regression models with fixed effects were used for the continuous variables (Medical expenditure) and logistic regression models with fixed effects were used for the categorical variables (Correct diagnosis, and Correct treatment).

Table S10 Association between patient-centered communication and primary care quality in year 2018

|  | Consultation time | | | | Number of unnecessary exams | | | | Number of unnecessary drugs | | | |
| --- | --- | --- | --- | --- | --- | --- | --- | --- | --- | --- | --- | --- |
|  | (1) | （2） | （3） | (4) | (5) | （6） | （7） | (8) | (9) | （10） | （11） | (12) |
|  | Coef.  (S.E.) | Coef.  (S.E.) | Coef.  (S.E.) | Coef.  (S.E.) | Coef.  (S.E.) | Coef.  (S.E.) | Coef.  (S.E.) | Coef.  (S.E.) | Coef.  (S.E.) | Coef.  (S.E.) | Coef.  (S.E.) | Coef.  (S.E.) |
| **PCC** | 0.27^***^  (0.05) |  |  |  | 0.002  (0.01) |  |  |  | 0.03^***^  (0.01) |  |  |  |
| **PCC1** |  | 0.45^***^  (0.08) |  |  |  | 0.02  (0.02) |  |  |  | 0.03^*^  (0.01) |  |  |
| **PCC2** |  |  | 2.13^***^  (0.60) |  |  |  | -0.07  (0.15) |  |  |  | 0.30^***^  (0.11) |  |
| **PCC3** |  |  |  | 0.33^***^  (0.09) |  |  |  | -0.02  (0.02) |  |  |  | 0.07^***^  (0.02) |
| Private | 0.06  (0.70) | -0.03  (0.64) | 0.35  (0.82) | 0.21  (0.76) | 0.46^*^  (0.24) | 0.45^*^  (0.25) | 0.46^*^  (0.24) | 0.46^*^  (0.24) | -0.17  (0.13) | -0.17  (0.13) | -0.14  (0.13) | -0.16  (0.14) |
| Non-alliance | -1.98^*^  (1.13) | -1.74  (1.21) | -1.36  (0.89) | -2.44^**^  (1.00) | 0.002  (0.30) | 0.03  (0.29) | -0.03  (0.31) | 0.01  (0.31) | 0.44  (0.28) | 0.44  (0.29) | 0.54^*^  (0.28) | 0.38  (0.29) |
| SP gender | -0.97  (1.14) | -0.92  (1.14) | -0.001  (1.17) | -0.85  (1.19) | 0.03  (0.34) | 0.01  (0.34) | 0.01  (0.35) | 0.05  (0.34) | -0.61^**^  (0.26) | -0.58^**^  (0.26) | -0.48^*^  (0.26) | -0.62^**^  (0.26) |
| 30-39 | -1.14^*^  (0.60) | -1.03^*^  (0.58) | -1.38^**^  (0.65) | -1.24^*^  (0.63) | -0.16  (0.16) | -0.15  (0.17) | -0.16  (0.16) | -0.16  (0.16) | 0.11  (0.12) | 0.12  (0.12) | 0.08  (0.12) | 0.10  (0.12) |
| 40-49 | -0.11  (1.38) | -0.29  (1.34) | -0.54  (1.21) | 0.15  (1.39) | 0.28  (0.25) | 0.27  (0.24) | 0.30  (0.26) | 0.27  (0.26) | 0.18  (0.17) | 0.17  (0.18) | 0.12  (0.15) | 0.23  (0.17) |
| ≥50 | 0.12  (1.38) | -0.10  (1.34) | -0.21  (1.20) | 0.28  (1.37) | 0.19  (0.23) | 0.18  (0.22) | 0.19  (0.24) | 0.17  (0.24) | 0.28  (0.17) | 0.26  (0.18) | 0.23  (0.16) | 0.32^*^  (0.18) |
| Physician gender | 0.54  (1.32) | 0.47  (1.27) | -0.10  (1.14) | 0.66  (1.37) | 0.28  (0.26) | 0.27  (0.25) | 0.29  (0.27) | 0.26  (0.27) | 0.18  (0.16) | 0.18  (0.17) | 0.09  (0.15) | 0.21  (0.17) |
| Year | 2.33^***^  (0.62) | 1.12  (0.68) | 1.73^***^  (0.64) | 2.91^***^  (0.66) | 0.60^***^  (0.15) | 0.57^***^  (0.16) | 0.60^***^  (0.15) | 0.52^***^  (0.17) | -0.02  (0.12) | -0.12  (0.12) | -0.08  (0.11) | 0.16  (0.16) |
| *N* | 244 | 244 | 244 | 244 | 244 | 244 | 244 | 244 | 244 | 244 | 244 | 244 |
| *R*^2^ | 0.36 | 0.36 | 0.32 | 0.31 | 0.22 | 0.22 | 0.22 | 0.22 | 0.19 | 0.17 | 0.19 | 0.20 |

Note: Standard errors in second column; ^*^ *p* < .1, ^**^ *p* < .05, ^***^ *p* < .01; (1) ~ (12) represents 12 different models; Ordinary least-squares regression models with fixed effects were used for the continuous variables (e.g., Consultation time, Number of unnecessary exams, Number of unnecessary drugs).

Table S11 Association between patient-centered communication and primary care quality by controlling different potential confounding factors

|  | Consultation time | | | | Number of unnecessary exams | | | | Number of unnecessary drugs | | | |
| --- | --- | --- | --- | --- | --- | --- | --- | --- | --- | --- | --- | --- |
|  | (1) | （2） | （3） | (4) | (5) | （6） | （7） | (8) | (9) | （10） | （11） | (12) |
|  | Coef.  (S.E.) | Coef.  (S.E.) | Coef.  (S.E.) | Coef.  (S.E.) | Coef.  (S.E.) | Coef.  (S.E.) | Coef.  (S.E.) | Coef.  (S.E.) | Coef.  (S.E.) | Coef.  (S.E.) | Coef.  (S.E.) | Coef.  (S.E.) |
| PCC | 0.09^*^  (0.05) |  |  |  | 0.02  (0.01) |  |  |  | 0.04^***^  (0.01) |  |  |  |
| PCC1 |  | 0.16^**^  (0.08) |  |  |  | 0.03  (0.02) |  |  |  | 0.03  (0.02) |  |  |
| PCC2 |  |  | 0.74^*^  (0.39) |  |  |  | 0.04  (0.11) |  |  |  | 0.34^***^  (0.11) |  |
| PCC3 |  |  |  | 0.09  (0.09) |  |  |  | 0.01  (0.02) |  |  |  | 0.07^***^  (0.02) |
| Private | 1.03  (0.83) | 0.82  (0.86) | 0.91  (0.83) | 1.14  (0.83) | 0.85^***^  (0.29) | 0.81^***^  (0.30) | 0.84^***^  (0.30) | 0.87^***^  (0.29) | -0.26  (0.17) | -0.30^*^  (0.17) | -0.31^*^  (0.16) | -0.16  (0.16) |
| Non-alliance | -3.48^**^  (1.72) | -3.51^**^  (1.71) | -3.54^**^  (1.62) | -3.78^**^  (1.75) | 0.02  (0.43) | 0.02  (0.43) | -0.04  (0.44) | -0.04  (0.43) | 0.12  (0.28) | 0.03  (0.26) | 0.13  (0.26) | 0.06  (0.24) |
| SP gender | -0.44  (0.67) | -0.39  (0.68) | -0.45  (0.73) | -0.45  (0.70) | 0.53^***^  (0.18) | 0.54^***^  (0.18) | 0.53^***^  (0.19) | 0.53^***^  (0.18) | -0.45^***^  (0.12) | -0.44^***^  (0.12) | -0.46^***^  (0.12) | -0.47^***^  (0.13) |
| 30-39 | 2.31  (1.55) | 2.25  (1.53) | 2.22  (1.45) | 2.44  (1.48) | 0.35  (0.35) | 0.33  (0.36) | 0.36  (0.38) | 0.37  (0.37) | -0.07  (0.21) | -0.06  (0.24) | -0.12  (0.23) | -0.02  (0.21) |
| 40-49 | 2.31  (1.60) | 2.30  (1.58) | 1.98  (1.49) | 2.39  (1.54) | 0.14  (0.35) | 0.13  (0.36) | 0.12  (0.38) | 0.15  (0.36) | -0.06  (0.24) | -0.05  (0.27) | -0.21  (0.25) | -0.01  (0.23) |
| ≥50 | 2.08  (1.68) | 2.09  (1.64) | 1.86  (1.53) | 2.22  (1.61) | -0.25  (0.35) | -0.26  (0.35) | -0.25  (0.37) | -0.23  (0.36) | 0.04  (0.22) | 0.07  (0.26) | -0.08  (0.25) | 0.08  (0.21) |
| Physician gender | -0.88  (0.77) | -0.80  (0.81) | -0.89  (0.78) | -0.97  (0.77) | -0.16  (0.18) | -0.14  (0.18) | -0.17  (0.18) | -0.17  (0.18) | 0.06  (0.24) | 0.06  (0.11) | 0.06  (0.10) | 0.01  (0.11) |
| Physician working experience | 0.69  (0.77) | 0.70  (0.78) | 0.60  (0.79) | 0.63  (0.77) | -0.11  (0.20) | -0.11  (0.20) | -0.13  (0.20) | -0.12  (0.20) | 0.04  (0.16) | 0.03  (0.16) | 0.01  (0.15) | 0.03  (0.16) |
| High school and above | -3.43^**^  (1.48) | -3.33^**^  (1.42) | -3.74^**^  (1.48) | -3.68^**^  (0.45) | 0.12  (0.39) | 0.15  (0.39) | 0.06  (0.38) | 0.07  (0.38) | -0.25  (0.38) | -0.30  (0.43) | -0.37  (0.40) | -0.32  (0.37) |
| Practicing (assistant) physician | 0.29  (0.87) | 0.26  (0.87) | 0.27  (0.93) | 0.10  (0.89) | 0.14  (0.24) | 0.14  (0.24) | 0.11  (0.23) | 0.11  (0.23) | 0.08  (0.15) | 0.02  (0.15) | 0.10  (0.15) | 0.04  (0.15) |
| Year | 0.91  (2.02) | 1.38  (2.01) | 0.57  (2.09) | 0.53  (2.13) | -0.54  (0.72) | -0.44  (0.70) | -0.59  (0.74) | -0.61  (0.75) | 0.22  (0.36) | 0.25  (0.34) | 0.08  (0.49) | 0.02  (0.37) |
| Case | 0.23  (0.61) | -0.10  (0.63) | 0.46  (0.63) | 0.51  (0.60) | 0.37^***^  (0.14) | 0.30^*^  (0.16) | 0.41^***^  (0.13) | 0.43^***^  (0.12) | 0.07  (0.10) | 0.05  (0.10) | 0.17  (0.11) | 0.22^*^  (0.12) |
| *N* | 239 | 239 | 239 | 239 | 239 | 239 | 239 | 239 | 239 | 239 | 239 | 239 |
| *R*^2^ | 0.26 | 0.26 | 0.25 | 0.25 | 0.29 | 0.29 | 0.28 | 0.28 | 0.26 | 0.21 | 0.26 | 0.27 |

Note: Standard errors in second column; ^*^ *p* < .1, ^**^ *p* < .05, ^***^ *p* < .01; (1) ~ (12) represents 12 different models; Ordinary least-squares regression models with fixed effects were used for the continuous variables (e.g., Consultation time, Number of unnecessary exams, Number of unnecessary drugs).

Table S12 Association between patient-centered communication and primary care quality for normalization

|  | Consultation time | | | | Number of unnecessary exams | | | | Number of unnecessary drugs | | | |
| --- | --- | --- | --- | --- | --- | --- | --- | --- | --- | --- | --- | --- |
|  | (1) | （2） | （3） | (4) | (5) | （6） | （7） | (8) | (9) | （10） | （11） | (12) |
|  | Coef.  (S.E.) | Coef.  (S.E.) | Coef.  (S.E.) | Coef.  (S.E.) | Coef.  (S.E.) | Coef.  (S.E.) | Coef.  (S.E.) | Coef.  (S.E.) | Coef.  (S.E.) | Coef.  (S.E.) | Coef.  (S.E.) | Coef.  (S.E.) |
| **PCC** | 1.07^***^ |  |  |  | 0.04  (0.05) |  |  |  | 0.21^***^  (0.04) |  |  |  |
| **PCC1** |  | 1.29^***^  (0.23) |  |  |  | 0.11^*^  (0.06) |  |  |  | 0.13^***^  (0.04) |  |  |
| **PCC2** |  |  | 1.01^***^  (0.22) |  |  |  | 0.03  (0.05) |  |  |  | 0.16^***^  (0.05) |  |
| **PCC3** |  |  |  | 0.45^**^  (0.20) |  |  |  | -0.03  (0.06) |  |  |  | 0.22^***^  (0.04) |
| Private | 0.19  (0.21) | 0.003  (0.60) | 0.24  (0.63) | 0.28  (0.65) | 0.35^**^  (0.15) | 0.33^**^  (0.15) | 0.35^**^  (0.14) | 0.35^**^  (0.14) | -0.14  (0.11) | -0.16  (0.11) | -0.13  (0.10) | -0.11  (0.10) |
| Non-alliance | -1.57^***^  (0.63) | -1.44^**^  (0.64) | -1.07^*^  (0.62) | -1.90^***^  (0.63) | -0.0004  (0.19) | 0.02  (0.18) | 0.01  (0.20) | -0.01  (0.21) | 0.36^*^  (0.18) | 0.34^*^  (0.17) | 0.42^**^  (0.17) | 0.28  (0.18) |
| SP gender | 0.09  (0.46) | 0.23  (0.45) | 0.16  (0.48) | 0.07  (0.48) | 0.35^**^  (0.15) | 0.36^**^  (0.15) | 0.35^**^  (0.15) | 0.36^**^  (0.15) | -0.23^**^  (0.11) | -0.21^*^  (0.11) | -0.22^*^  (0.11) | -0.26^**^  (0.11) |
| 30-39 | -0.42  (0.44) | -0.34  (0.45) | -0.41  (0.44) | -0.45  (0.45) | -0.09  (0.11) | -0.08  (0.11) | -0.09  (0.11) | -0.08  (0.11) | 0.02  (0.08) | 0.03  (0.08) | 0.03  (0.08) | 0.01  (0.08) |
| 40-49 | -0.95  (1.10) | -0.92  (1.07) | -1.08  (0.99) | -0.83  (1.10) | 0.35^*^  (0.18) | 0.35^*^  (0.18) | 0.35^*^  (0.18) | 0.36^*^  (0.19) | 0.01  (0.13) | 0.03  (0.15) | -0.005  (0.13) | 0.02  (0.14) |
| ≥50 | -0.86  (1.07) | -0.89  (1.05) | -1.16  (0.95) | -0.80  (1.07) | 0.29  (0.18) | 0.29  (0.18) | 0.28  (0.18) | 0.29  (0.19) | 0.02  (0.13) | 0.02  (0.15) | -0.03  (0.13) | 0.04  (0.14) |
| Physician gender | -0.69  (1.09) | -0.61  (1.05) | -1.13  (0.95) | -0.73  (1.10) | 0.22  (0.19) | 0.23  (0.18) | 0.21  (0.19) | 0.22  (0.19) | 0.09  (0.13) | 0.09  (0.15) | 0.02  (0.14) | 0.08  (0.14) |
| Year | 1.56  (1.34) | 2.02  (1.34) | 0.15  (1.45) | 0.66  (1.48) | 0.39  (0.48) | 0.48  (0.48) | 0.34  (0.48) | 0.33  (0.19) | 0.02  (0.26) | -0.05  (0.27) | -0.25  (0.32) | -0.10  (0.27) |
| Case | 0.70  (0.42) | 0.05  (0.47) | 0.77^*^  (0.41) | 1.10^***^  (0.39) | 0.53^***^  (0.09) | 0.47^***^  (0.10) | 0.53^***^  (0.09) | 0.52^***^  (0.47) | 0.05  (0.07) | 0.01  (0.08) | 0.07  (0.07) | 0.19^**^  (0.08) |
| *N* | 492 | 492 | 492 | 492 | 492 | 492 | 492 | 492 | 492 | 492 | 492 | 492 |
| *R*^2^ | 0.26 | 0.27 | 0.26 | 0.22 | 0.19 | 0.19 | 0.19 | 0.19 | 0.18 | 0.14 | 0.16 | 0.19 |

Note: Standard errors in second column; ^*^ *p* < .1, ^**^ *p* < .05, ^***^ *p* < .01; (1) ~ (12) represents 12 different models; Ordinary least-squares regression models with fixed effects were used for the continuous variables (e.g., Consultation time, Number of unnecessary exams, Number of unnecessary drugs).
